# Photonics of human saliva: potential optical methods for the screening of abnormal health conditions and infections

**DOI:** 10.1007/s12551-021-00807-8

**Published:** 2021-06-02

**Authors:** Jijo Lukose, Sanoop Pavithran M., Mithun N., Ajaya Kumar Barik, Keerthilatha M. Pai, V. K. Unnikrishnan, Sajan D. George, V. B. Kartha, Santhosh Chidangil

**Affiliations:** 1 Centre of Excellence for Biophotonics, Department of Atomic and Molecular Physics, Manipal Academy of Higher Education, Manipal, Karnataka 576104 India; 2 Department of Oral Medicine and Radiology, Manipal College of Dental Sciences, Manipal, Manipal Academy of Higher Education, Manipal, Karnataka 576104 India; 3 Centre for Applied Nanoscience, Department of Atomic and Molecular Physics, Manipal Academy of Higher Education, Manipal, Karnataka 576104 India

**Keywords:** Photonics, Saliva, COVID-19, SPR, Raman spectroscopy, SERS, FTIR, HPLC-LIF

## Abstract

Human saliva can be treated as a pool of biological markers able to reflect on the state of personal health. Recent years have witnessed an increase in the use of optical devices for the analysis of body fluids. Several groups have carried out studies investigating the potential of saliva as a non-invasive and reliable clinical specimen for use in medical diagnostics. This brief review aims to highlight the optical technologies, mainly surface plasmon resonance (SPR), Raman, and Fourier transform infrared (FTIR) spectroscopy, which are being used for the probing of saliva for diverse biomedical applications. Advances in bio photonics offer the promise of unambiguous, objective and fast detection of abnormal health conditions and viral infections (such as COVID-19) from the analysis of saliva.

## Introduction

Here we review well-recognized photonics-based technologies, surface plasmon resonance (SPR), Raman, and Infrared spectroscopy, which are being used currently for analysing clinical samples like body fluids (saliva, blood, urine and vaginal wash), biopsy tissue samples, and cellular samples in diverse biomedical applications, and discuss how these photonics-based methods applied to saliva can be a highly promising technology for universal screening in situations like the present COVID-19 pandemic.

## Photonics-based technologies and their significance

Though photonics techniques can involve the entire range of the electromagnetic spectrum-from gamma rays to microwaves (wavelengths of fraction of a nanometer to several cms)-current interest on bio-medical applications is mostly centred in the UV-Visible-Infra red region, covering optical radiation in the range of a few hundred nanometres to about a hundred microns (10^6^ to10^2^ cm^-1^ (wave numbers)), and we will cover only this range in the present review. Powerful portable/miniature radiation sources like tunable lasers, super radiant light sources, light-emitting diodes (LEDs), and laser-driven light sources (LDLSs) are now commercially available. New techniques like broadband optical coherent emission has also been shown to be capable of direct IR spectra measurement, opening up possibility of broadband infrared spectroscopic study of samples with sensitivity down to submicrograms/ millilitre in samples like serum (Pupeza et al. 2020).

Till a little more than a decade back, spectroscopic systems involved relatively large instrumentation, making them amenable to only lab-based operation. Miniature spectrometers are now available as off-the-shelf units. Portable/handheld spectroscopic/photonics-based instrumentation, like mobile-phone-based examination systems, are available now^.^ Also optical fibre technologies have transformed telecommunication by enabling big data transfer with reduced latency. These advances have facilitated the use of photonics-based instrumentation by trained technicians, for survey of cases in “As Is, Where Is” conditions, and transmission of the data to centrally located facilities with qualified medical professionals for decision making (Cai et al. 2017; Rani et al. 2019; Ko et al. 2020; Pal et al. 2020). The combination of miniature systems, like mini-detectors with mini-spectrometers have made it possible at present to use of techniques like multi-directional functional spectroscopy systems for imaging applications on proof of concept (POC) basis (Shimokawa et al. 2019). Combination of these advances in instrumentation, data transmission and data processing, with artificial intelligence (AI) and machine learning (ML) processes have made photonics-based technologies easily adaptable for universal health-care applications, involving large population groups spread across several location (Belushkin et al. 2020; Nogueira 2020).

Optical spectroscopy techniques have proved to be of great utility in providing novel solutions in many clinical needs (Al-Muslet and Ali 2011; Popp et al. 2011; Spyratou et al. 2012; Krafft 2016; Cordero et al. 2018; Wallace 2019). These include, the use of optical coherence tomography in ophthalmic care, photonics based medical imaging for preclinical research, Raman, fluorescence and other spectroscopic studies of clinical samples, and observation of disease-specific markers at ultra-trace levels, all of which allow regular screening, early detection, tracking and follow-up in many diseases, both communicable and non-communicable. Contactless, non-invasive photonic-based wearable sensors, which can provide rapid, and precise health information are also becoming common-place now (Yang and Gao 2019). Most important, photonics-based methods like Laser-induced fluorescence -LIF-, laser raman spectroscopy, photo thermal spectroscopy, hyperspectral imaging, laser-induced breakdown spectroscopy (LIBS), and reflectance spectroscopy are capable of remote, non-contact observation, and hence are highly suitable for identification of harmful or abnormal conditions in any kind of samples, including, clinical, environmental, and other (e.g., human and animal subjects) samples by remote, non-contact observations (Shameem et al. 2017; Bishop et al. 2019; Gabbarini et al. 2019; Mandrile et al. 2019; Nganou et al. 2019; Yeh et al. 2020).

## Potential of saliva as a unique clinical specimen

**Fig. 1:**
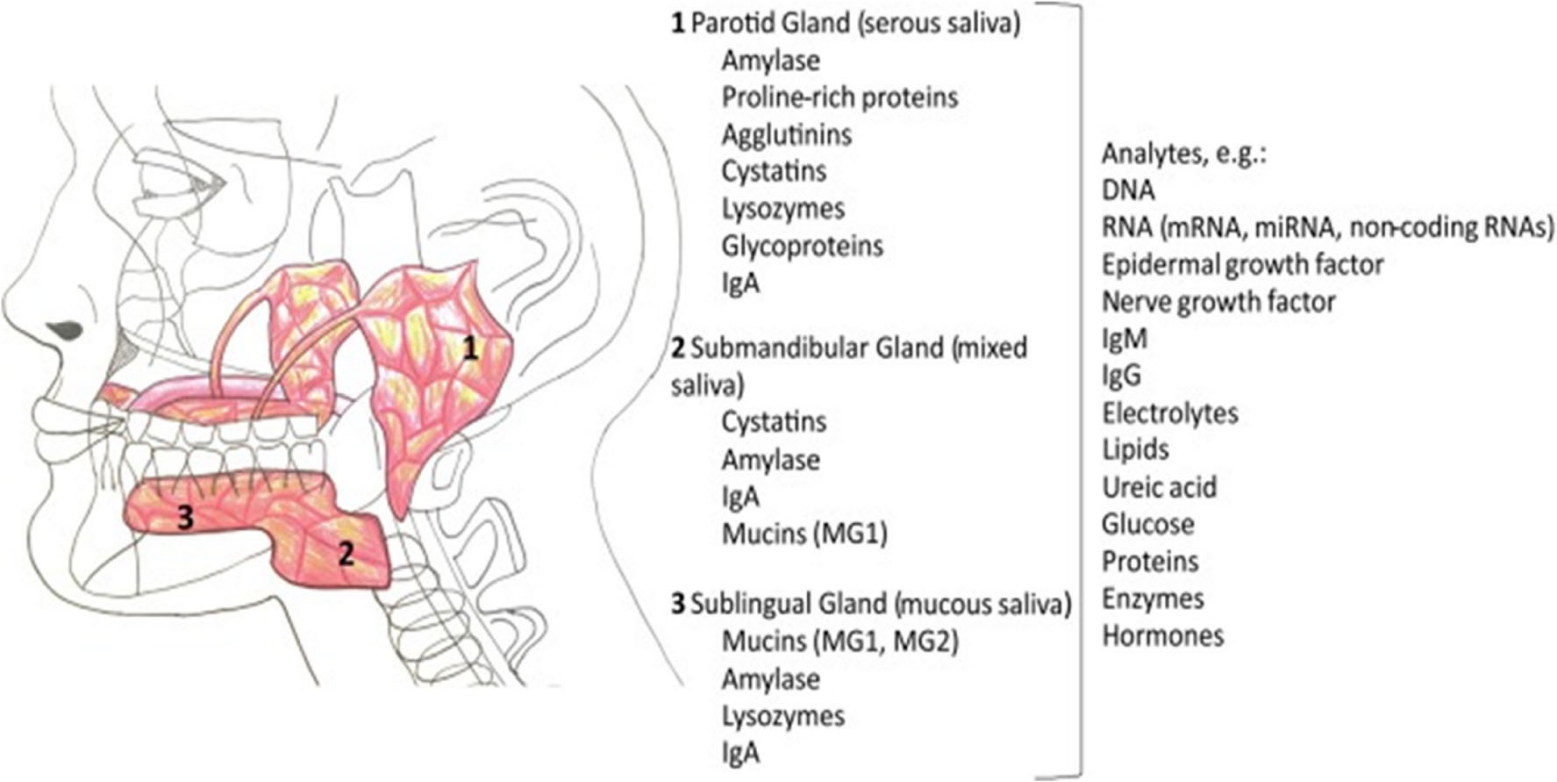
Schematic illustration of the major salivary glands and their contributions to the components of saliva. Reprinted with permission from (Roblegg et al. 2019)

Saliva is a transparent hypotonic aqueous solution produced by the salivary glands (Fig. 1). It acts as a detergent of the teeth and oral cavity, and as a lubricant for food ingestion. A healthy adult produces ~ 600 ml saliva in a day, having a pH range 6.6–7.1 (Zhang et al. 2016). Around 99% of saliva comprises of water and a variety of inorganic ions (e.g. K^+^ , Na^+^ , Ca^2+^, Mg^2+^, Cl^−^ , HCO_3_^−^ , H_2_PO_4_^−^ , HPO_4_^2−^ ), proteins (immunoglobulins, enzymes), mucus, urea, and uric acid forming the remaining portion (Bonifacio et al. 2015). In addition, more than 700 microorganisms have also been reported in saliva which can be linked with oral health and other systemic diseases (Zhang et al. 2016). It has been suggested that the human oral microbiome contributes more than 2000 microbial proteins from more than fifty bacterial genera to the saliva proteome (Katsani and Sakellari 2019). Salivary biomarkers have been suggested as highly suitable for regular screening and early detection of many abnormal conditions (Farah et al. 2018; Liu et al. 2018; Shah 2018; Gug et al. 2019; Pathiyil and Udayasankar 2019).

The growing appeal in saliva research for clinical applications has resulted in the appearance of various devices in market which facilitates both sterile and safer saliva collection (Khurshid et al. 2016)^.^ Non-invasive screening and early diagnosis of diseases, especially viral infections, from human saliva utilizing optical tools can be a reliable option for mass screening, especially in situations like COVID-19, where sampling and analysis of several hundreds of samples will be required fast with minimum contact with the subjects/sample. Unlike blood and throat swab, saliva is easier to collect, store and transport (Wang et al. 2016; Ilea et al. 2019). Sample collection procedure does not demand the requirement of an expert in phlebotomy as in case of blood collection (Azzi et al. 2020). This can also minimize the risk of exposing health/social workers to blood-borne diseases (Kaczor-Urbanowicz et al. 2017). Since the sample collection procedure is painless, it will be more acceptable for haemophiliacs, neonates, elderly, and disabled people (Guilbault et al. 1995; Malon et al. 2014). This will be also an added advantage in increasing the compliance/cooperation from the suspected/vulnerable cases who need recurrent clinical monitoring via multiple sampling over the day or several days. Self-collection of samples by vulnerable subjects itself can undo direct interaction between patient and healthcare worker, which can be the primary risk factor for nosocomial infections (To et al. 2019; Ceron et al. 2020).

In this review, we will try to cover the various photonics tools which can be used for probing saliva samples. Being label free and highly sensitive tools, the core part of the review is confined to the studies regarding the exploration of plasmonic, infrared, raman, and laser-induced fluorescence approaches for the analysis of salivary fluids. In the current threatening pandemic scenario, it may be highly useful if the many research centres, health agencies, and health care providers explore the various photonics-based diagnostics opportunities which can lead to fast, universal screening and detection techniques.

## Optical spectroscopy techniques for saliva

### Surface plasmon resonance

Surface plasmon resonance technique has received much attention recently for biomedical applications, owing to its high sensitivity. The technique offers label free detection of biological analytes without the requirement of any fluorescing or radioactive tags. Surface plasmons are guided electron oscillations confined to a thin layer of a metal-dielectric interface (Liedberg et al. 1995). Excitation of surface plasmons can be realized using an incident radiation, once the optical wave vector parallel to the interface matches the wave vector of surface plasmon. Under optimal conditions, optically excited surface plasmon resonance (SPR) could be quite strong and a large portion of optical energy is dissipated into a guided electromagnetic wave along the interface leading to absorption of energy. As the extent of energy transfer is highly sensitive to the coupling conditions, parameters like the refractive index of the dielectric layer can be accurately determined by monitoring the reflected light intensity or phase, which can be explored for various bio-sensing applications (Homola 2003; Homola and Piliarik 2006; Lukose et al. 2016; Lukose et al. 2018). Adsorption of any target sample on the sensor surface using surface functionalized receptors can induce a refractive index variation, which can be identified by tracking the change in the conditions of the resonance coupling of incident light to the propagating surface Plasmon wave (SPW). Resonance coupling can be identified by a dip in the reflectivity of the light spectrum, which is traditionally tracked by measuring the wavelength, the incident angle or the intensity of the reflected light.

**Fig. 2:**
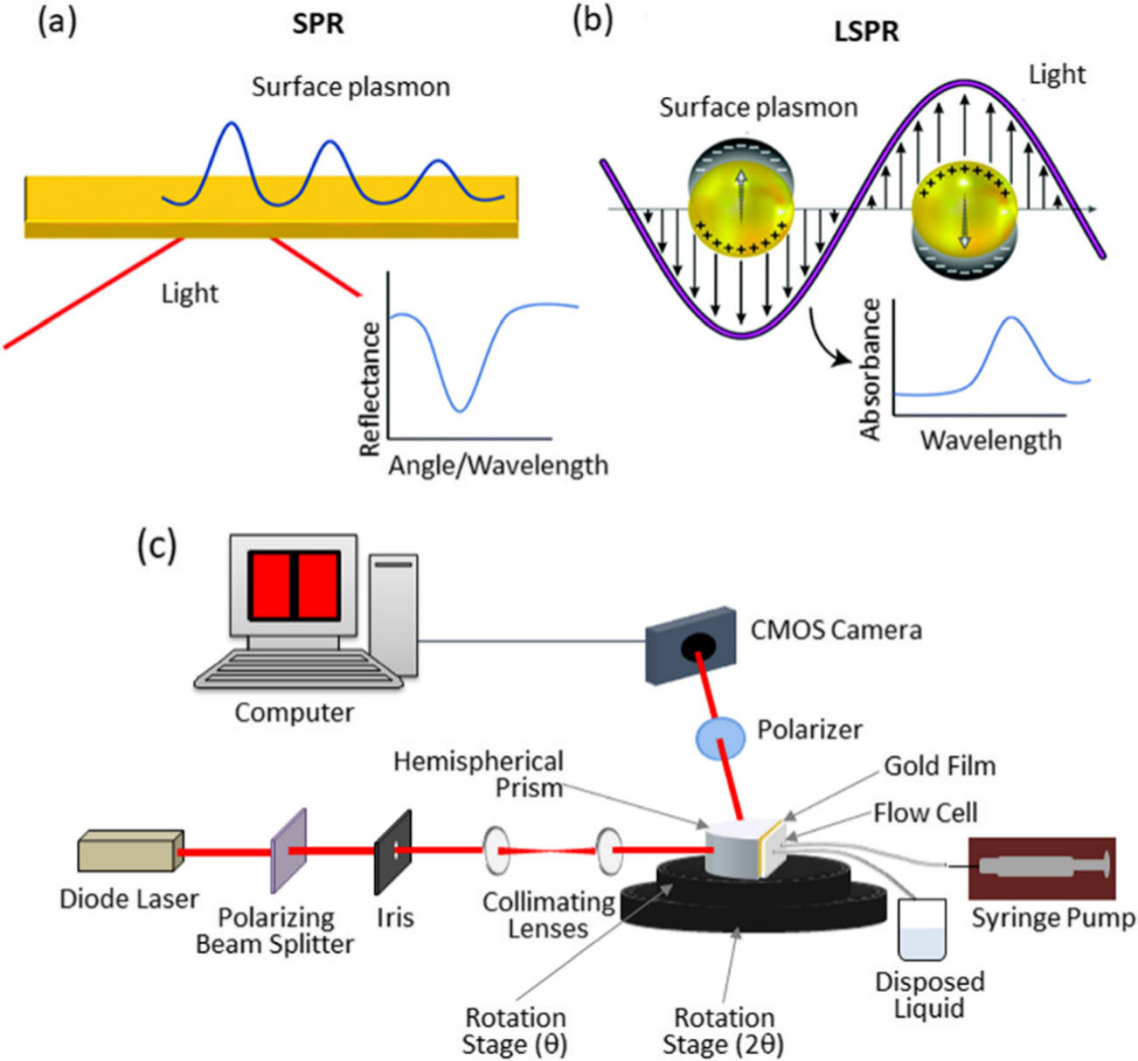
A) Prism coupling configuration of SPR, where a light beam impinges on a thin metallic film deposited on a prism. P-polarized light absorbed by the surface plasmon is seen from a minimum in the reflection spectra. B) Representation of the localized surface plasmon on nanoparticles and absorbance spectra obtained for binding events on nanoparticles. Reproduced from (Masson 2020) with permission from The Royal Society of Chemistry. (b) SPRi instrument developed for biosensing. Reproduced from (Lukose et al. 2018)

Recently this technique has been employed to study the interaction between ACE-2 receptor and spike glycoprotein of coronavirus (SARS-COV, SARS-COV-2, MERS-COV) (Shang et al. 2020; Wang et al. 2020). SARS-COV-2 have shown comparatively higher binding affinity towards ACE-2 receptor as per the SPR sensorgram results. Highly localized effect is observed, once the plasmonic resonance is restricted to a nanomaterial surface instead of a planar substrate, leading to a non-propagating localized surface plasmon with a specific frequency, which is termed as localized surface plasmon resonance (LSPR) (Fig. 2a) ( Wang et al. 2020). Plasmonic-based sensors have also been widely exploited for the detection of analytes in human saliva and to monitor the interactions of salivary proteins.

LSPR has been used to study cortisol (indicator for stress), Cathepsin and Mucin (Fernández-González et al. 2007; Stevens et al. 2008; Frasconi et al. 2009; Mitchell et al. 2009; Gorodkiewicz and Regulska 2010; Gorodkiewicz et al. 2011; Tahara et al. 2014; Jo et al. 2020). Gorodkiewicz et al have conducted Cathepsin G (CatG) detection in saliva using a custom built SPR imaging instrument (Gorodkiewicz et al. 2011). Perturbations of CatG activity in saliva have a critical role in the pathobiochemistry and diagnostics of salivary gland, gingiva, and oral mucosa diseases (Ozmeric 2004). SPR imaging (SPRi) technique has been employed for the determination of cystatin (a marker for chronic renal failure) in human body fluids such as plasma, urine and saliva (Gorodkiewicz and Luszczyn 2011; Alsamarai et al. 2018). LSPR has also been used for the detection of drugs – cocaine, MDMA (3,4-methylenedioxymethamphetamine) and fenetoyin^.^ in saliva (Liu and Delgado 1999; Fu et al. 2007; Sonny et al. 2010). Simple laboratory-built LSPR systems (Fig. 2.b) have been used for real-time, rapid detection of various biomolecules and pathogens (Lukose et al. 2016).

**Fig. 3:**
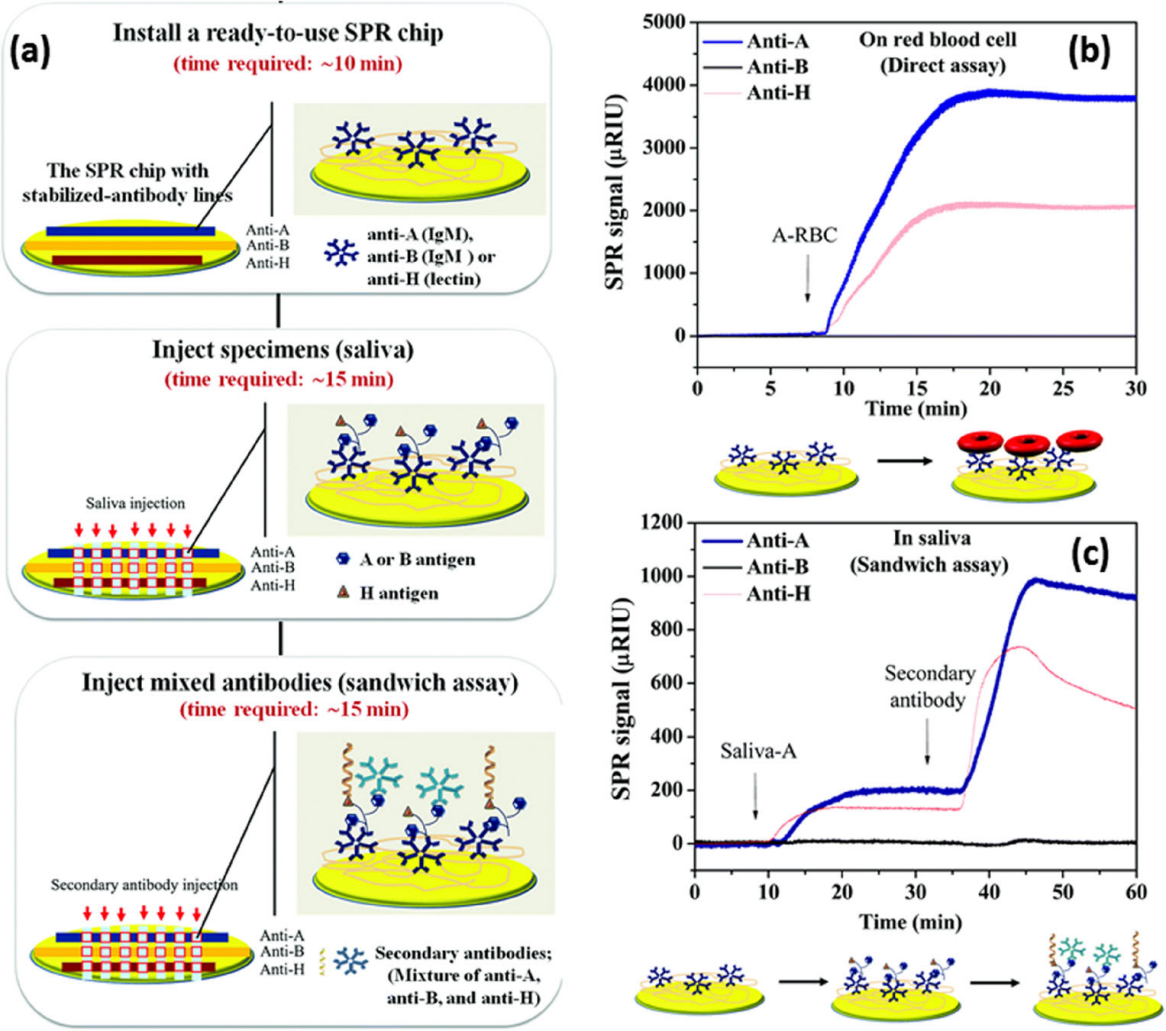
(a) SPR procedure for ABH antigen detection in saliva. (b, c) SPR sensorgrams of ABH antigen detection in red blood cells (direct assay) (b) and saliva (sandwich assay) (c). Reproduced from Ref. (Peungthum et al. 2017)

Peungthum et al. have combined SPR imaging with antibody array method for the quantitative detection of ABH antigens in saliva (Peungthum et al. 2017). As given in the Fig. 3, a multiplex format consisting of an antibody array with immobilized anti-A, anti-B and anti-H on the hydrogel-coated surface was developed to specifically quantify A, B and H substances simultaneously. A sandwich assay with a mixture of anti-A, anti-B and anti-H antibodies was developed to increase sensitivity. The assay demonstrated good specificity and precision when diluting saliva specimens (Fig. 3). SPR technique using sandwich immunoassay has shown high specificity is less complicated than the usual neutralization agglutination test which demands skilled personal, is time consuming, and susceptible to operator interpretation error. Musso et al. have coupled SPRi with mass spectrometry for the analysis of protein biomarkers in human saliva (Musso et al. 2015). SPR technique has been used to monitor the kinetic interactions between Porphyromonas gingivalis fimbriae and various salivary proteins in comparison with haemoglobin and fibrinogen (Amano et al. 1999). Guerreiro et al have a developed a biosensor comprising SPR and molecular imprinted polymers (MIP) for evaluating the interactions between saliva and polyphenol compounds for wine astringency estimation (Guerreiro et al. 2017). The astringency obtained using this technique over a range of wine samples have shown good correlation with the values estimated from a trained sensorial panel, which suggests the utility of this method as a quantitative approach during wine production.

**Fig. 4:**
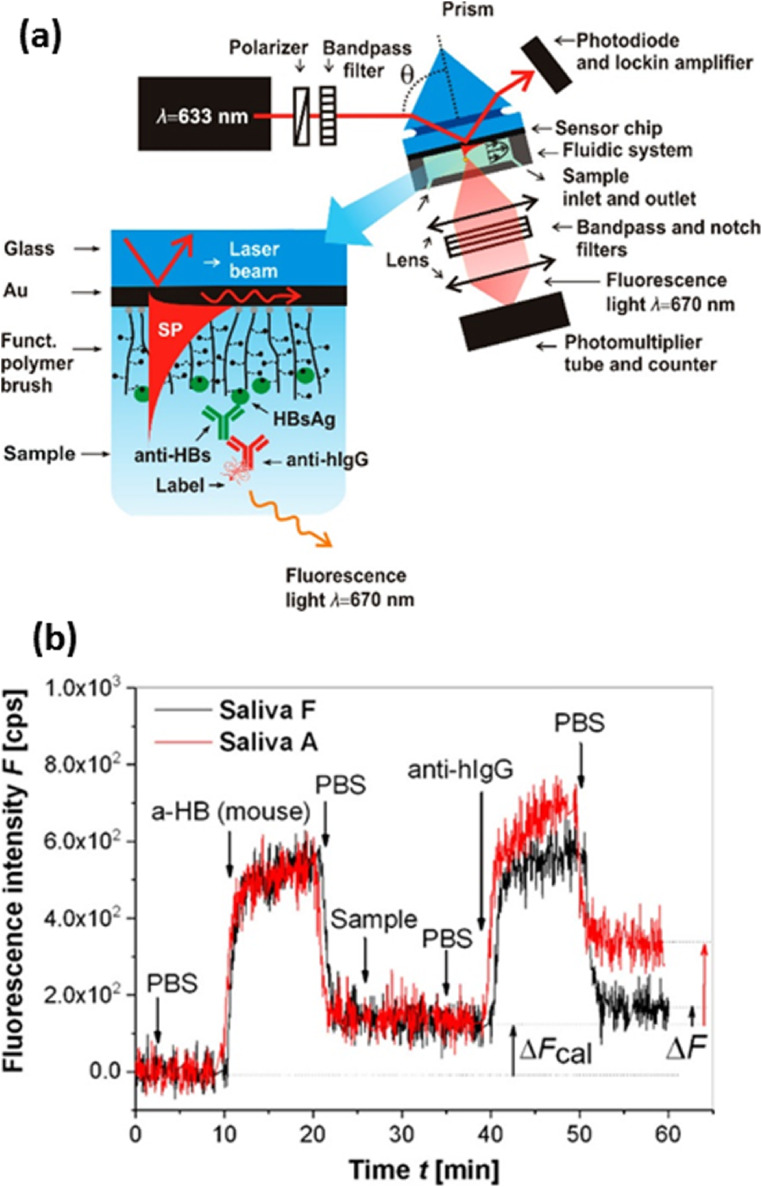
(a). Schematics of plasmon-enhanced fluorescence spectroscopy biosensor with a detail of sensor chip with poly(HPMA-co-CBMAA) brush functioning as a binding matrix. (b). Example of kinetics of fluorescence signal for negative and highly positive saliva samples. Reproduced under a Creative Commons Attribution (CC-BY) License from (Riedel et al. 2017)

Researchers have also used LSPR technique to monitor the salivary pH (Luo et al. 2017). Saliva pH is an indicator for evaluating oral health conditions as well as diseases such as mucosal stomatitis which show lower pH value. Some of the drug activities also relies on pH of saliva. Yang et al. have performed quantitative measurements of interleukin-8 (IL-8) protein in saliva in view of reports about the potential of salivary IL-8 level as a biomarker for oropharyngeal squamous cell carcinoma (OSCC) (Yang et al. 2005). Liang et al. have demonstrated the detection of carbohydrate antigen 15-3 (CA15-3), a biomarker for the early detection of breast cancer, in saliva (Liang et al. 2012). In another study, SPR method was utilized for matrixmetalloproteinase-9 (MMP-9) detection, which is of interest in chronic periodontitis (CP) disease. High levels of MMP-9 (above 20 ng/ml) in human saliva can be an indicator for CP condition (Isaza-Guzmán et al. 2011). MMP-9 detection with an LOD of 8 pg/ml was achieved using anti-MMP-9 immobilized on carboxymethyldextran (CM-5) sensor chip. Concentration of MMP in saliva collected from normal subjects were found to be lower than those of periodontitis patients, as was seen from the SPR measurements (Mohseni et al. 2016). Using aptamer functionalized surfaces, SPR biosensor has been used for detection of avian influenza virus in poultry swab samples of saliva in the range of concentrations 0.128 to 12.8 HAU (haemagglutination units) (Bai et al. 2012). Dostalek et al. have developed a novel plasmonic enhanced fluorescence biosensor for the hepatitis B detection from saliva samples (Riedel et al. 2017). Anti-HBs antibodies from clinical samples was detected using the combination of angular interrogation SPR and plasmonically enhanced fluorescence (Fig. 4). The sensor response obtained for saliva collected from volunteers who showed positive (saliva A) and negative response (saliva F) during ELISA test of serum samples is given in Fig. 4(b).

### Raman and IR spectroscopy

**Fig. 5:**
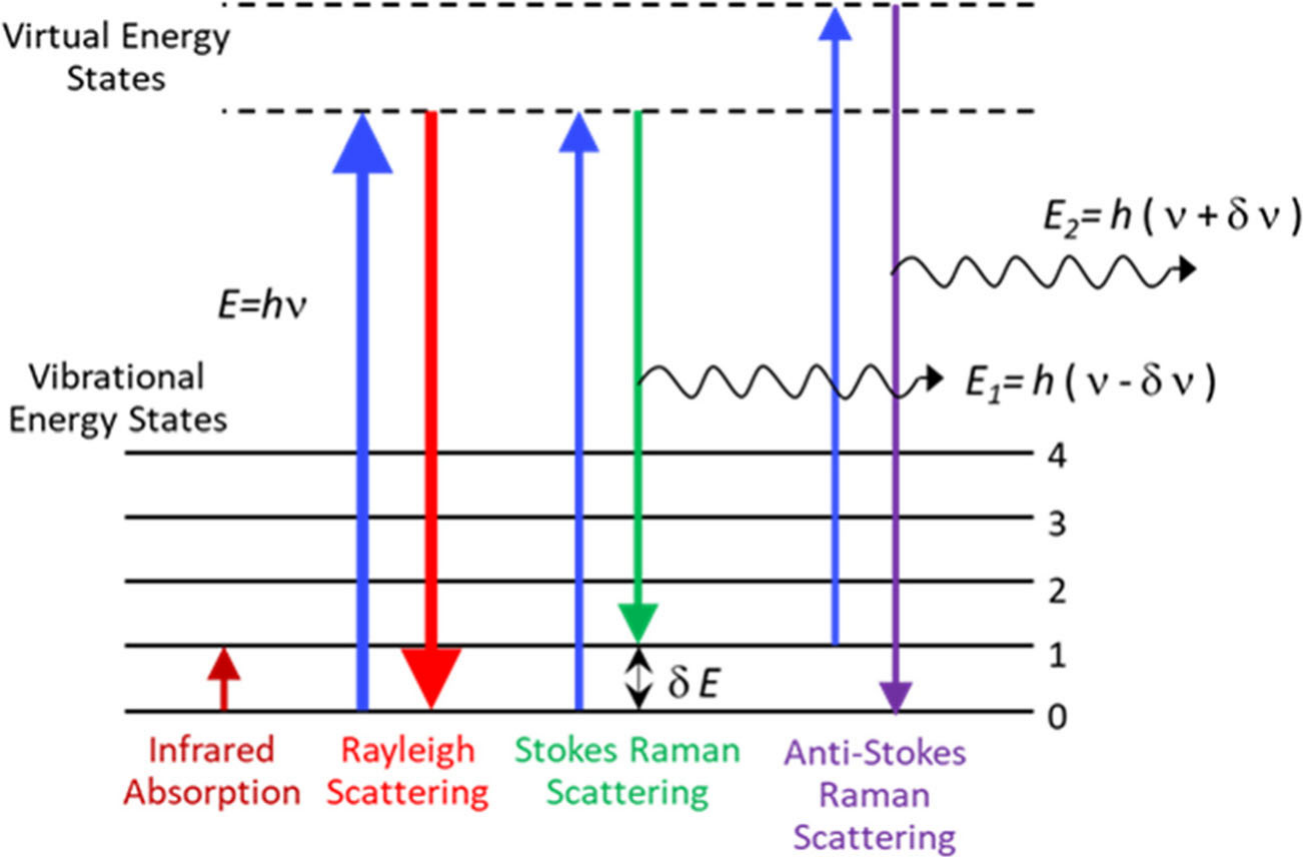
Vibrational energy level diagram of a molecular system with Raman scattering process and infrared absorption

Vibrational spectroscopic techniques, infra-red (IR) absorption/reflection, and Raman scattering, depend on the interaction of electromagnetic radiation with the dipole moment or polarizability, respectively, of a molecule, leading to changes in the vibrational-rotational energy states. This interaction between the radiation and molecular species is measured by observing the changes in the spectral distribution of the incident radiation in the interaction, namely absorption or scattering. In IR spectroscopy one usually measures the absorption or reflection of mid Infrared radiation by the sample. In Raman scattering, the sample is excited with radiation at a chosen wavelength and the spectral distribution of the scattered radiation is measured to determine the energy lost/gained from vibrational energy level changes during the interaction. The basic schematic diagram of Raman scattering and IR absorption is depicted in Fig. 5.

Since the energy levels of a molecule are determined by the structure of the molecule, which are highly specific for the molecule concerned, information on the individual molecules and their amounts present in the sample can be derived from IR and Raman spectra. Advances in instrumentation, radiation sources, and data processing techniques at present, have made these techniques capable of fast, routine measurements on many types of samples. Moreover, the non-destructive nature of the technique and the requirement of only micro amounts of samples have made these techniques highly useful for clinical applications (Baker and Faulds 2016).

### Raman spectroscopy of saliva

Raman spectroscopy of clinical samples like body fluids (e.g., blood, saliva, urine), biopsy tissue and cellular samples can provide information about the sample, highly useful in clinical diagnostics, pharmaceutical sciences, drug abuse and forensic applications (Sikirzhytski et al. 2012; Han et al. 2015; Zapata et al. 2015; Bunaciu et al. 2017; Parlatan et al. 2019). Raman spectroscopic investigations of saliva samples for forensic purposes have been reported by a number of groups. Differentiation of body fluids such as peripheral blood, saliva, semen, sweat, and vaginal fluid has been demonstrated with ~100% accuracy by using multivariate analysis of the Raman spectroscopy data (Muro et al. 2016a, 2016b). Sex determination from dry saliva has been performed with more than 90 % accuracy (Muro et al. 2016a, 2016b). In a similar work, the spectra of dried saliva obtained from multiple donors have shown consistent bands derived from proteins, an acetate, a saccharide, and the amino acid, arginine (Virkler and Lednev 2010).

The inherent low cross-sections for Raman scattering have somewhat restricted its use for clinical applications requiring trace detection and high sensitivity. However, this drawback can be overcome with the use of two special techniques, surface enhanced Raman scattering (SERS), and resonance Raman scattering (RRS), which provide signal enhancement by several orders of magnitude. SERS is capable of detection down to single molecule levels (enhancement of intensities to even by an order of 10^14^), while resonance Raman has been reported to give enhancements up to 10^6^ times the signal observed in conventional Raman spectroscopy, due to both the resonance effect, and the dependence of scattering cross sections on 1/ λ^4^, where λ is the excitation wavelength (Langer et al. 2019). The resonance Raman spectra, especially for molecules like proteins, and nucleic acids are observed at wavelengths of the order of 250nm, while conventional Raman for biological systems uses NIR (>750nm) excitation to avoid strong background fluorescence (Kneipp et al. 1998; Wood et al. 2005; Tuschel 2018).

**Fig. 6:**
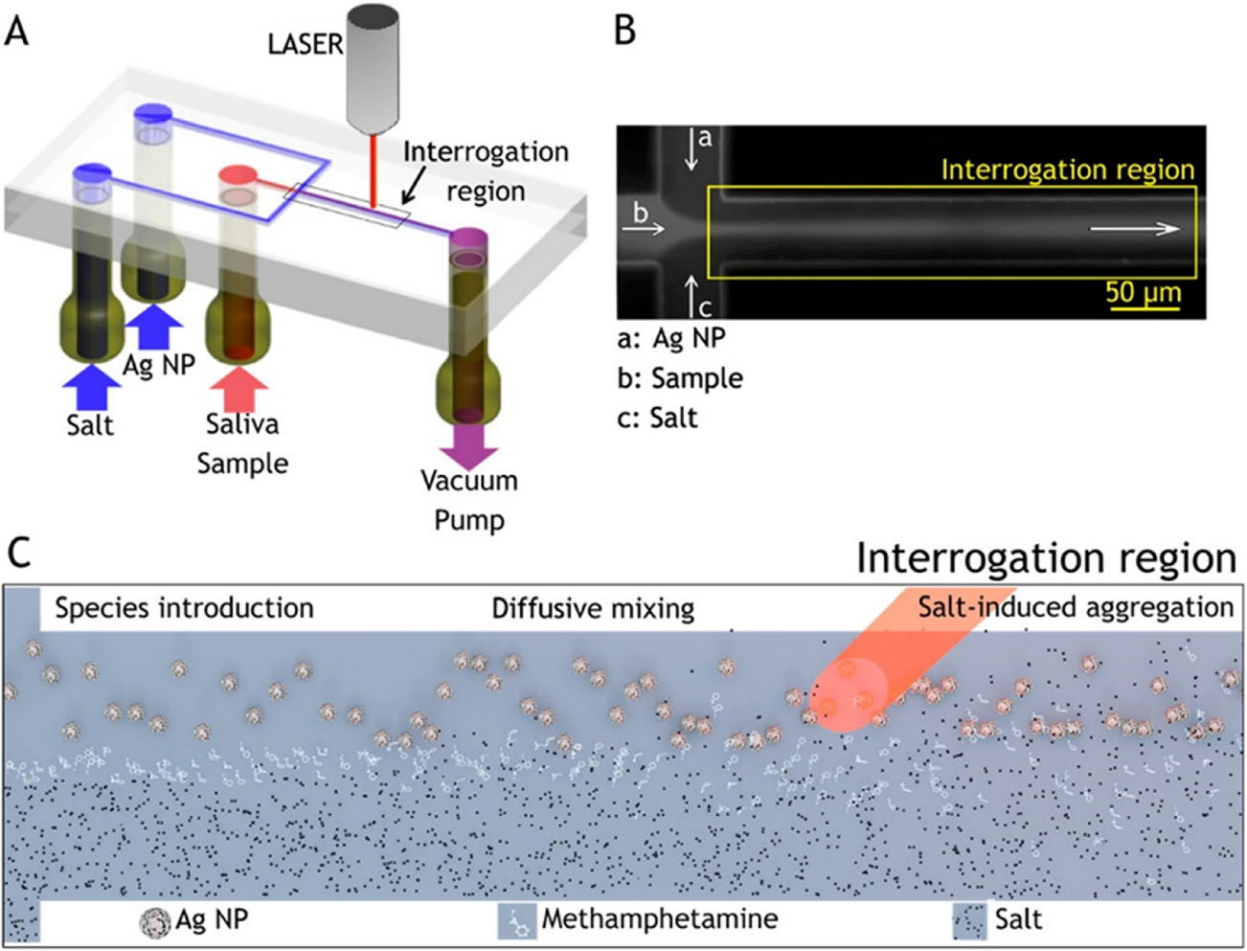
Flow-focusing microfluidic device used for controlled Ag-NP aggregation. (A) Ag-NP suspension, a saliva sample, and salt solution are loaded in the device and driven through it by a vacuum pump. (B) At the flow-focusing junction, the sample stream is enveloped by the side-streams and diffusion drives lateral mass transport between the laminar flows, here visualized with a fluorescent dye. (C) Schematic of the reaction: Ag NP, analyte and salt solution are introduced to the channel from the left and flow toward the right. Analyte molecules resident in the focused stream diffuse laterally into the side flows. Salt ions also diffuse into the colloid stream inducing controlled nanoparticle aggregation, creating SERS-active clusters that convect downstream. Interrogating the region rich in colloid dimers, which provide intense plasmonic enhancement, we are able to achieve optimal SERS-based detection. Reprinted from (Andreou et al. 2013) Copyright (2013) American Chemical Society

SERS has been used for the identification of drugs in saliva. Dana et al. used gold and silver sol gels as SERS probes to detect cocaine in saliva at clinical concentrations, as low as 25 ng/ml (Dana et al. 2015). SERS technique using metal-doped sol-gels was employed for the identification of drugs as well as metabolites (caffeine, phenobarbital, cocaine, amphetamine, diazepam, methadone, and 1-(1-phenylcyclohexyl) piperidine (PCP)) present in human saliva (Shende et al. 2005; Inscore et al. 2011). Magnetic assay-based SERS technique was also reported, in which Au nanoparticles-doped magnetic nanocomposites (AMN), modified with inositol hexakisphosphate substrate, was used for generating enhanced Raman signal of trace amounts of drug-related biomarkers in saliva (Yang et al. 2015). Magnetically induced SERS assay have displayed two Raman bands at 1030 cm^-1^ and 1052 cm^-1^ for cotinine in saliva, whereas only a weak band at 1030 cm^-1^ was observed in routine SERS assay. SERS technique was also adopted for developing portable/field usable devices for the detection of illicit drug presence (diazepam) in saliva in ~15 min (Shende et al. 2014). SERS strips which can be inserted into a portable Raman spectrometer have been used in field applications for the rapid measurement of codeine and fentanyl in saliva (Shende et al. 2019). SERS measurements conducted on a group of narcotic users have displayed an additional band at 1030 cm^-1^ in their saliva with respect to non-narcotic users (Anyu et al. 2009). Quantitative measurement of heroin, morphine monohydrate (MM), morphine-3-β-glucuronide (M3B), and monoacetyl morphine (6MAM) in saliva samples using SERS was reported recently (Akçan et al. 2020). SERS detection of trace levels of cocaine in raw saliva was achieved without any sample pre-processing. Inscore et al. studied identification of certain illicit drugs in saliva with SERS, including heroin, without measuring their concentrations (Farquharson et al. 2011; Inscore et al. 2011). Andreou et al have performed the detection of illicit drugs in saliva with a micro-fluidic device, which exploits the SERS technique induced by the aggregation of AgNPs as shown in Figure 6. (Andreou et al. 2013). A laminar flow composing a stream of methamphetamine along with two sheath streams of salt solutions and AgNPs were produced inside the channel. At the interrogation region, SERS-active nanoparticle dimers and small-order aggregates with methamphetamine predominantly formed. Due to the low affinity of methamphetamine to the silver nanoparticles, the salt was added to induce the aggregation. Trace concentrations of methamphetamine in saliva were detected within minutes. The microfluidic sensor does not demands any additional chemical functionalization and reactants requirements for drug detection.

Cocaine detection at trace levels as low as 10ng/ml was reported using gold nanorods synthesized using seed-mediated, surfactant assisted method (D’Elia et al. 2018). Silver doped sol-gel SERS substrates were used to detect 5-fluorouracil, chemotherapeutic drug spiked in saliva at physiological range of concentrations (Farquharson et al. 2005). The same group have extended similar studies on 5-fluorouridine and 5-fluoro-2’-deoxyuridine too (Farquharson et al. 2008). Silver nano flowers formed on a paper substrate was successfully employed for SERS as well as ambient pressure mass spectrometry analysis aimed at the detection of Ketoprofen in dried saliva (Díaz-Liñán et al. 2020).

In another work, microfluidic SERS platform, created using homogeneous plasmonic mirror like capsules, has been utilized for the detection of the illicit drug, methamphetamine (MA) in urine and human saliva (Su et al. 2019). Su et al. could identify the specific vibrational fingerprint band at 993 cm^-1^ in all the samples containing MA in ppm concentration, which was not observed in blank saliva. Detection of the primary component of marijuana, tetrahydrocannabinol (THC) in body fluids plays a critical role in forensic analysis and public health. Trace level detection of THC from saliva was reported using hybrid plasmonic biosilica nanomaterials by depositing silver nanoparticles (Ag NPs) into diatom photonic, where the band at 1621 cm^−1^ due to the O-C=O stretching in THC, could be observed (Sivashanmugan et al. 2019a, b). The group have extended the study and developed diatomaceous SERS substrates based protocol and detected THC using a portable device (Sivashanmugan et al. 2019a, b). Optoplasmonic materials are of high interest in SERS substrate fabrication due to their unique properties of comprising both photonic and plasmonic elements. A layer of analyte-carrying dielectric nano/micro spheres is placed on top of a monolayer of gold nanoparticles to enhance the intensity of the electric (E-) field localization and to enrich the analyte close to the electromagnetic hot spots. Surface receptors immobilized on these dielectric microsphere enable the binding of target analytes in liquid samples. Deposition of the analyte loaded microspheres on the self-assembled gold nanoparticle ensures a high local concentration of analytes in the electromagnetic “hot” surface. Optoplasmonic SERS substrates have been found effective in the detection of methamphetamine in saliva and urine at nanomolar concentrations (Hong et al. 2020). Li et al. have developed an immunochromatographic assay (ICA) based on SERS technique for detecting morphine, which is the main metabolic end product of opium and heroin. The detection limit of 2.4 × 10^−4^ng/ml has been obtained for opium by this method, which is significantly lower than traditional ICA method (Li et al. 2020). Authors have proposed this method as a rapid morphine detection technique for the screening.

SERS technique of saliva has a high potential in clinical applications also. Lung cancer diagnosis from saliva was reported with SERS, using silver hydrosols. PCA-LDA analyses of the SERS spectra were able to discriminate lung cancer group from the control, where the major contributions were found to be due to the decrease in proteins and nucleic acid for cancerous patients (Li et al. 2011). SERS study was reported for the first time on saliva from patients with Sjögren's Syndrome (SjS) and validated with two-dimensional shear wave elastography data obtained from parotid glands. Combination of SERS spectral results with elastography studies have provided the sensitivity and specificity of 80% and 81% respectively (Moisoiu et al. 2020). SERS technique was also employed as a non-invasive tool for the diagnosis of oral cancer from saliva. Thiocyanate, which is regarded as biomarker for human health and smoking status of individuals, has been detected in saliva from the SERS spectra. The band at 2126 cm^-1^ from C-N stretch of thiocyanate has been found higher in cancer subjects (Fălămaș et al. 2020). Similar results have been obtained in study on oral dysplasia samples also (Daniel et al. 2020). SERS technique has been proposed for oral cancer diagnosis by evaluating the S100 calcium-binding protein P (S100P) present in saliva (Han et al. 2019). The results indicated a threefold increase of S100P in oral cancer patients as compared to healthy subjects^.^ Qiu et al. detected biochemical differences in the saliva of subjects with nasopharyngeal carcinoma when compared with healthy subjects with a diagnostic sensitivity of 86.7%, specificity of 81.3% and accuracy of 83.9% (Qiu et al. 2016). Sialic acid (SA) determination in saliva can provide clues towards early diagnosis of breast cancer. Colloidal solution of citrate reduced silver nanoparticles as SERS substrates mixed with saliva collected from breast cancer patients have displayed a SA concentration of 18.5 ± 9.7 mg/dL which is significantly higher compared to the 3.5 ± 1.0 mg/dL in controls (Hernández-Arteaga et al. 2017). Hernández-Cedillo et al. used SERS method for the Sialic acid (SA) level determination in a study performed on 93 subjects which comprised of three classes, control, subjects with periodontitis and gingivitis (Hernández-Cedillo et al. 2019). The study found elevated levels of SA in periodontitis subjects in citrate reduced silver nanoparticles mixed with saliva. Monitoring of urease activity in saliva can be useful for the screening of helicobacter pylori infection and to prevent dental caries (Zhang et al. 2017). It is well-known that urea gets converted to NH_3_ and CO_2_ in the presence of urease. SERS peak due to urea present at 1003 cm^−1^ can be used to detect the presence of urease in oral cavity fluid. An intensity decline in this band was observed in saliva spiked with urease (Hu et al. 2019). Zamora-Mendoza et al. have carried out SERS-based non-invasive diagnosis of asthma by identifying biomarkers in saliva. Multivariate analysis of the spectral data has shown a sensitivity of 85%, specificity of 82% and an accuracy of 84% for asthma diagnosis in children. The band at 1326 cm^-1^ from interleukin 8 (IL-8) has shown statistically significant differences between asthma patients and control group in addition to the increase in characteristic bands of lipids in asthma subjects (Zamora-Mendoza et al. 2019). Cao et al. have showed that SERS may be a possible tool for the detection of acute myocardial infarction (AMI) from saliva (Cao et al. 2015). PCA-LDA analysis performed on the spectral data acquired from the saliva of normal and AMI patients have a diagnostic sensitivity and specificity of 80.4% and 81.4%, respectively.

In a recent study, Žukovskaja et al. have demonstrated Pyocyanin metabolite detection in saliva using SERS based microfluidic biosensor at a detection limit of 10 μM. Pyocyanin is a metabolite specific for the pathogen Pseudomonas aeruginosa, which is the etiological agent for respiratory tract infections (Žukovskaja et al. 2017). Eom et al. have reported the utility of SERS technique for the detection of oseltamivir-resistant pH1N1/H275Y mutant virus in saliva (Eom et al. 2019). Oseltamivirhexylthiol (OHT), viral receptor functionalized on gold nanoparticles has been able to detect mutant viral at 1 PFU concentration. Sun et al. have developed a magnetic SERS immunesensor for detecting avian influenza virus (AIV) from saliva using a portable device (Sun et al. 2017). SERS combined with machine learning algorithms has been reported for the determination of non-structural protein (NS1) present in saliva, which can be critical in early diagnosis of dengue fever (Othman et al. 2019). Wasik et al. detected the presence of the non-structural protein (NS1) in the adulterated artificial human saliva over the range of clinically relevant concentrations using SERS technique as an early detection procedure for dengue fever (Wasik et al. 2018). In a very recent work, SERS technique was suggested for the detection of SARS-Cov-2 from saliva in a preliminary study. Spectral differences have been identified in the gold nanoparticles mixed saliva spiked with S protein (Jinglin et al. 2020). Viral detection from saliva has been proposed in a recent study, where they were able to extract Raman signatures attributed to viral RNA (Desai et al. 2020). The group of Yacaman, who had previously developed the saliva-based SERS technique for breast cancer detection has come out with a new test for SARS-COV-2, by the SERS technique (Hernández-Arteaga et al. 2017; Yacaman et al. 2020).

### IR spectroscopy of saliva

The mid-IR (4000–400 cm^−1^) region is the region of main interest for biomedical applications. This comprises of the so-called fingerprint region, exhibiting characteristic absorption peaks for lipids, proteins, amide I/II, carbohydrates, and nucleic acids (Balan et al. 2019; Su and Lee 2020). Similar to Raman spectroscopy, Infrared spectroscopy provides “whole biochemical fingerprinting” of samples by means of the spectral features. IR spectroscopy can be performed using either the traditional, “direct transmission” mode or the surface sensitive ATR- (Attenuated Total Reflection) mode. Bio-medical studies mostly involve samples in the condensed phase (solids and liquids) and the individual absorption bands have half-widths 5–10 cm^-1^ at room temperatures. This allows the use of even miniature, portable Fourier transform (FT) and/or dispersive spectrometers for spectral measurements (Ayerden et al. 2014; Kruzelecky et al. 2017; Chai et al. 2020). FTIR gives highly accurate spectral data and have high sensitivity because of the multiplexing effect of total signal detection at any time, rather than single wavelength measurements as in diffraction spectrometers. The high accuracy of FTIR provides, precise frequency data, allowing very accurate spectral data processing like spectral subtraction and curve fitting can give information which can discriminate between even closely similar spectra of related species. Hence, FTIR spectroscopy is a very effective tool for bio-medical applications.

Takamura et al. have reported the ATR-FTIR spectra of five body fluids- peripheral blood, saliva, semen, urine and sweat (Takamura et al. 2018). The study coupled with chemometric analysis has been able to discriminate the body fluid types from the spectra, which is useful for forensic applications. Saliva spectra has shown characteristic peaks arising from proteins at 3279 cm^−1^ (Amide A), 1640 cm^−1^ (Amide I), 1537 cm^−1^ (Amide II) and 1239 cm^−1^ (Amide III). The symmetric and asymmetric C–H stretching and carbonyl (C=O) stretch of ester groups contributed to peaks at 2943 cm^−1^, 2854 cm^−1^, and 1743 cm^−1^. In addition, the thiocyanate characteristic band has been identified at 2057 cm^−1^. Quinn et al. have also used ATR-FTIR spectra for the discrimination of body fluids, for forensic applications (Quinn and Elkins 2017).

**Fig. 7:**
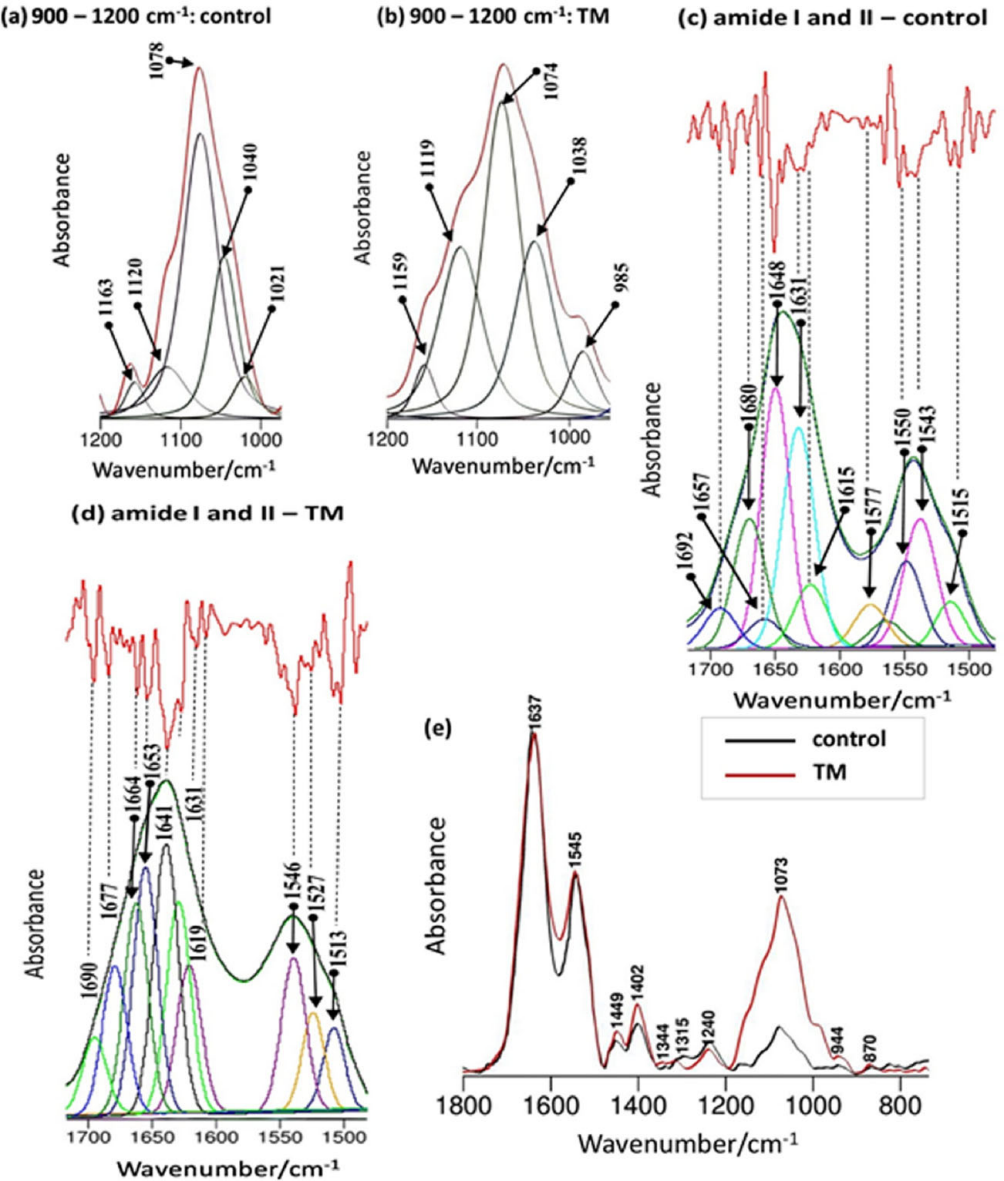
Curve-fitting analysis of the 900–1200 cm−1 spectral interval (a—control group, b—TM patients) and amide I/II with second derivative spectra (c—control group, d—TM patients) profiles together with (e) averaged ATR-FTIR spectra of saliva samples (black line—control group, red line—TM patients). Spectra were averaged from five healthy volunteers and five salivary gland tumour patients spectra, respectively. Reprinted from (Paluszkiewicz et al. 2020)

In a recent study, ATR-FTIR characterization of saliva has been found helpful in the diagnosis of chronic kidney disease (CKD). It has been found that the spectral markers of thiocyanate and phospholipids/carbohydrates can be a valuable biomarker panel for identifying CKD (Rodrigues et al. 2019a, b). Amide II band present at 1545 cm^-1^ in the ATR-FTIR spectra of saliva has been reported to be a characteristic marker for Psoriatic and Diabetic patients (Bottoni et al. 2015). A recent work has explored this technique in the diagnosis of periodontitis from saliva (Beyer-Hans et al. 2020). PCA analysis performed on ATR spectra could differentiate between controls and generalized aggressive periodontitis patients. Other studies have been reported on the differentiation of aggressive and chronic periodontitis using ATR-FTIR spectroscopy. These studies have identified differentiating features in 2800–3000 cm^-1^ assigned to lipids and proteins in periodontitis patients (Derruau et al. 2019). ATR-FTIR spectroscopic investigation of saliva has also been exploited to monitor the physiological stress in human body (Khaustova et al. 2010; Caetano Júnior et al. 2016). Ferreira et al. have recently shown the possibility of using IR spectra of saliva for non-invasive breast cancer detection. The authors have suggested two characteristic bands, 1041 cm^− 1^ in the second-derivative spectra and the 1433–1302.9 cm^− 1^ region of the original spectra for effective identification of benign and cancerous subjects from control (Ferreira et al. 2020). ATR spectroscopy has been employed for monitoring the biochemical variations in saliva collected from salivary gland tumour patients (Fig. 7) (Paluszkiewicz et al. 2020). Increase in phosphate-associated band at ~ 1074 cm^-1^ has been found during tumour progression in these subjects. In addition, an increase in β-sheet band at 1543 cm^-1^ and decrease in α-helix peak frequency around 1634–1640 cm^−1^ region has also been observed for the tumour samples. Clear alterations in secondary structure of proteins were evident in the saliva of tumour samples (Paluszkiewicz et al. 2020). Progesterone level variations during each trimester of pregnancy in normal and diabetic women have been observed in the saliva samples of corresponding subjects (Sultana et al. 2011).

Rodrigues et al. studied FTIR spectral patterns of saliva collected from females suffering from burning mouth syndrome (BMS), and enhancement of nucleic acid and thiocyanate bands has been found in these with respect to healthy females (Rodrigues et al. 2019a, b). Thiocyanate increase has been attributed due to greater proliferation of microorganisms in the saliva. Chances of cell necrosis have been speculated from the increase in DNA bands in the infrared spectra of saliva from chronic smokers (Rodrigues et al. 2017). Collagen content increase has also been found comparatively high in saliva of former smokers than chronic smokers, which indicated the improvement in inflammation tissue regeneration capacity after quitting smoking. FTIR spectroscopy of saliva has been used for the early detection of neonatal sepsis, which has been one of the primary cause for global neonatal mortality (Yunanto et al. 2019). Changes in DNA and protein composition have been evident from the spectra, which suggested protein damage in neonates struggling from sepsis infection. Recent years have witnessed high research interest in exosomes generated in urine, blood, and saliva, for clinical applications (Krafft et al. 2017, Mihály et al. 2017, Kim et al. 2018, Chiang and Chen 2019, Kim et al. 2019a, b, Yap et al. 2019). Salivary exosomes collected from oral cancer patients and healthy subjects have been evaluated in a recent work, where PCA-LDA (principal component analysis-linear discriminant analysis) could effectively discriminate them with a sensitivity, specificity and accuracy of 100%, 89%, and 95%, respectively (Zlotogorski-Hurvitz et al. 2019). In addition to the clinical diagnostics applications, IR spectroscopy has also been used for detecting drug abuse from IR spectra of human saliva. Hans et al. carried out ATR-FTIR investigation of saliva to obtain spectral features of cocaine at around 1280 cm^−1^ and between 1760 and 1710 cm^−1^ with an LOD of ~10μg/ml (Hans et al. 2012). An extended study reported by the same group has obtained a lower limit of ~1μg/ml (Hans et al. 2014).

### Other methods

**Fig. 8:**
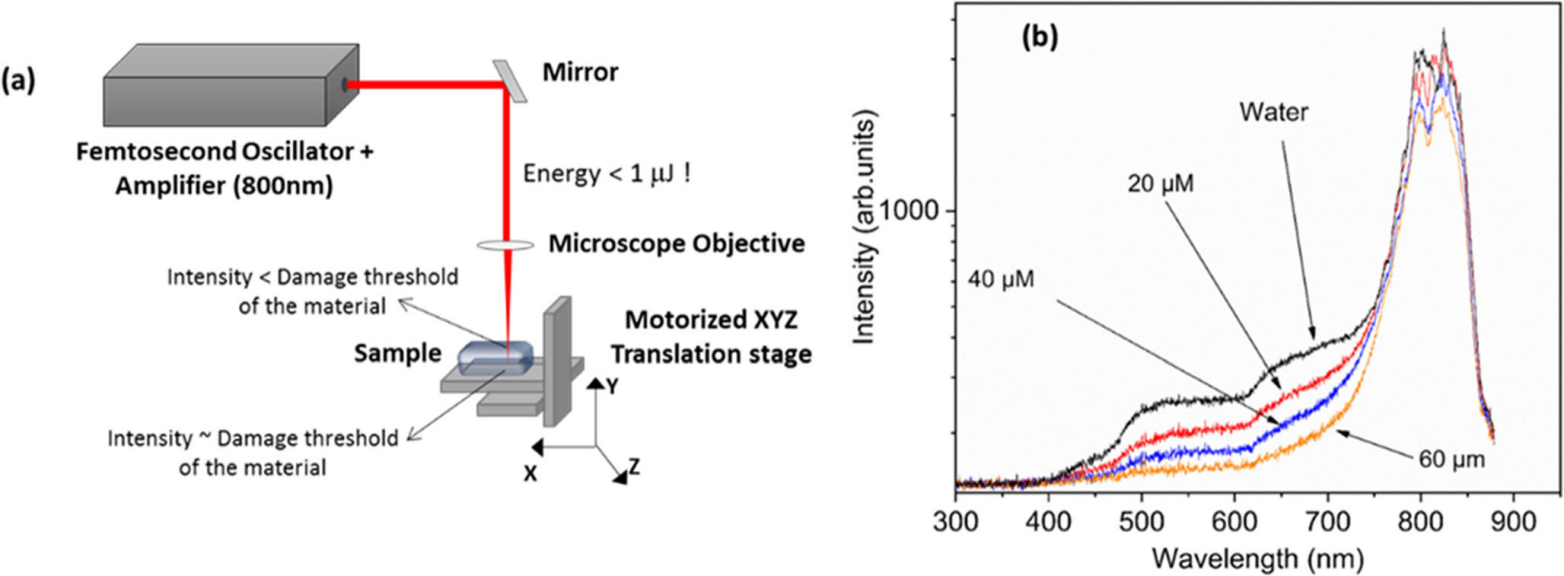
(a) Schematic of the experimental set up and (b) White light spectrum of pure H_2_O and H_2_O + α-amylase samples. Reproduced from (Chidangil et al. 2007) under Creative Commons (CC BY 4.0)

In addition to plasmonics and vibrational spectroscopic techniques, other approaches including or combined with optical spectroscopy have also been used for saliva based research. Intense ultrashort infrared laser light propagation through matter can cause supercontinuum white light generation, in which transmitted light spectra cover a much broader spectral range, compared to incident light. Our group have earlier conducted ultrafast supercontinuum generation studies in water using a femtosecond Ti-sapphire laser (820-nm and duration <45 fs ) in presence of salivary proteins (Chidangil et al. 2007). It has been observed that the addition of salivary protein α-amylase resulted in suppressing the supercontinuum generation in water, as shown in Fig. 8. Electron scavenging property of proteins has been identified as the rationale for this suppression. The experiments conducted on other proteins such as immunoglobulin, lysine, and arginine didn’t cause any suppression, which indicates the potential of this technique for sensing the stress marker α-amylase in saliva.

In a recent work, a label-free microfluidic-optoelectronic sensing platform for Point-Of-Care (POC) detection of stress biomarkers (cortisol, serotonin, dopamine, norepinephrine, and neuropeptide) in human body fluids, including saliva, has been developed. The study relies on the absorption characteristics of these biomarkers which lies in the near ultraviolet region of electromagnetic spectra (Ray and Steckl 2019). Yuvaraj et al. have conducted fluorescence-based investigation of saliva for oral cancer detection (Yuvaraj et al. 2014). The study targeted the auto fluorescence from salivary porphyrin excited at 405 nm, which has shown increase in its emission characteristics at 627 and 687 nm with respect to normal cases. In addition, the presence of flavins emission has been also observed as a small hump at 510 nm in malignant saliva. A fluorescence biosensor for ultrasensitive detection of oral cancer biomarker, Interleukin-8 (IL-8) protein in saliva has been also reported (Tan et al. 2008). Pradhan et al. have utilized stokes shift spectroscopy as a potential technique for the detection of oral malignancy from saliva (Kumar et al. 2018). Farago et al. have recently developed a fibre optic fluorescence sensor for the detection of blood in saliva (Farago et al. 2019). Coumarin based Fluorescent probes have been used for the detection of hydrogen sulphide in human saliva (Zaorska et al. 2018).

**Fig. 9:**
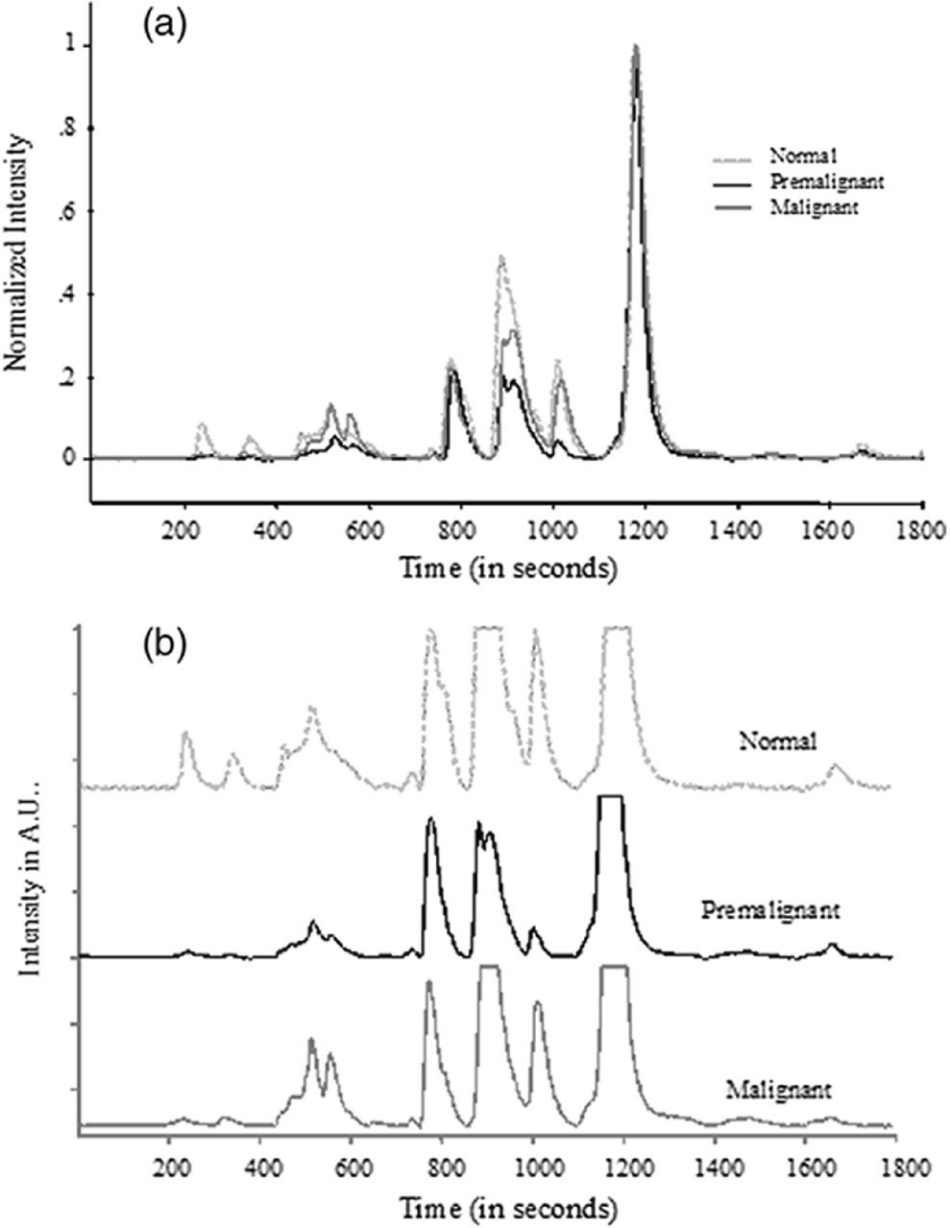
(a) Typical pre-processed saliva chromatograms of normal, premalignant, and malignant classes (normalized with respect to 1200 s peak). (b) Expanded (five times) chromatograms in (a). Reprinted from (Patil et al. 2013)

At present, markers for many diseases, like various cancers, are mostly detected by immunochemical methods like “ELISA” and Radio Immuno Assay. These techniques are time consuming, require prior knowledge of which antigen/antibody you are looking for, and so can be used only for specific proteins. High performance liquid chromatography-laser induced fluorescence (HPLC-LIF) technique has been used for ultrasensitive protein profiling of clinical samples like body fluids (blood, saliva, vaginal wash), tissue homogenates, and cellular lysates, for screening and early detection of oral, cervical, breast and ovarian cancer (Patil et al. 2013; Patil et al. 2015). Protein profiles recorded from the saliva of normal, premalignant and malignant oral cancer patients (Fig. 9) were analysed with statistical pattern analysis methods like PCA, and discrimination of normal, pre-malignant and malignant conditions has been achieved with high specificity and sensitivity (Patil et al. 2015). The protein profiling by HPLC-LIF has many advantages like very high sensitivity (subfemto-mole levels), no need of prior knowledge of the identity of the marker proteins for a given disease condition, and relatively fast, few tens of minutes.

**Fig. 10:**
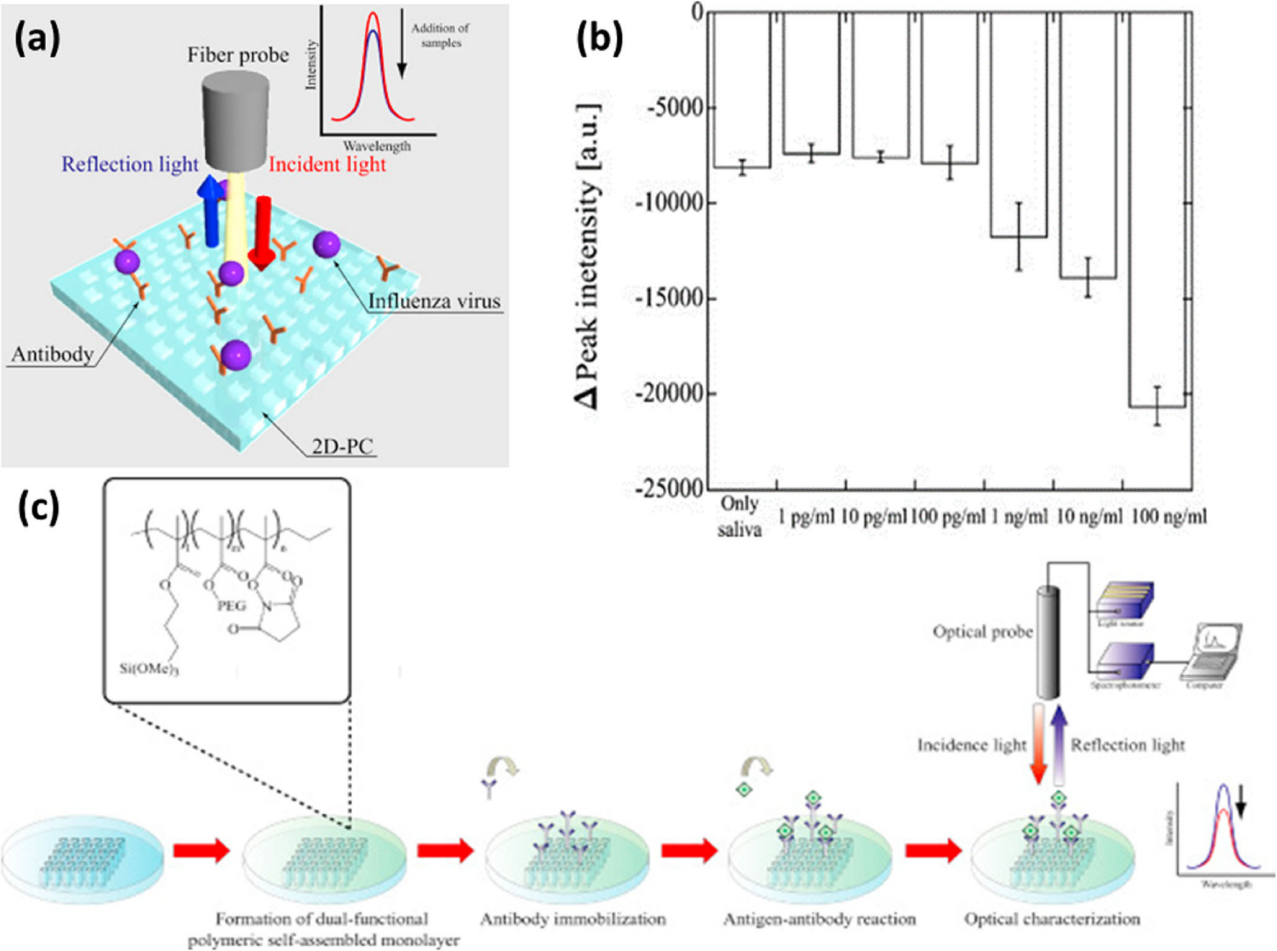
(a) Schematic illustration of reflectometric detection principle for influenza virus in human saliva using nanoimprint lithography (NIL)-based two-dimensional (2D) photonic crystal (PC). (b) Experimental procedure for the reflectometric detection of influenza virus in human saliva using antibody-immobilized 2D-PC. Reprinted from (Endo et al. 2010)

Mass spectrometry coupled with HPLC also has been used for the identification of salivary biomarkers for clinical diagnosis (Bigler et al. 2009). Laser induced breakdown spectroscopic study on saliva from smokers has witnessed an elevated calcium concentration, due to demineralisation (solubility) process which is related to the dissolution of Ca element in tooth lattice into saliva (Zahroh et al. 2019). A.K. Singh et al. have recently reported a handheld optical device which can be used for self-monitoring of glucose level in saliva (Singh and Jha 2019) .The enzyme glucose oxidase functionalized on the sensing strip along with a pH sensitive dye produces an enzymatic reaction followed by pH change upon glucose addition, which will be detected. A portable fluorescence based sensor has been developed for the detection of lithium in saliva excited at 590 nm wavelength, in which the emission at 620nm due to quinizarin-lithum ions interaction is monitored. The device has demonstrated a linear detection range of 0.25 ~ 6.0 mM of Li^+^ in saliva, which is the range of interest for therapeutic applications (Kim et al. 2011). Endo et al. has reported reflectometry based detection of influenza virus in saliva. Detection of influenza from saliva was carried out by evaluating the reflectance changes with respect to viral concentrations using antibody immobilized on a nanoimprint lithography based two dimensional photonic crystal biosensor (Fig. 10) (Endo et al. 2010).

Convat, a research team based at the Catalan Institute of Nanoscience and Nanotechnology, Spain, has recently announced a laser based nano-interferometric biosensor, which is claimed to be useful for real time and highly specific early diagnosis of COVID-19 from saliva. The sensor relies on the refractive index variation due to attachment of viral molecules on bioreceptors immobilized on sensor surface, which alter the direction in which light propagates. The distortion in this propagation direction is monitored for rapid viral diagnosis (Wallace 2020). In a recent work, J. Wang et al. have developed dual-functional plasmonic biosensors which comprise the plasmonic photothermal (PPT) effect and localized surface plasmon resonance (LSPR) sensing transduction for the detection of the selected sequence from SARS-CoV-2 (Qiu et al. 2020). Two dimensional gold nanoislands (AuNIs) functionalized with complementary DNA receptors were employed for the detection of SARS-CoV-2 through nucleic acid hybridization. The thermoplasmonic heating generated by the gold nanoislands chip via illumination at plasmonic resonance frequency found to elevate the in-situ hybridization temperature and facilitate the accurate discrimination of two similar gene sequences. The limit of the detection of the dual-functional LSPR sensors for the selected SARS-CoV-2 was estimated to be 0.22 pM. The developed technique to provide real-time, label-free detection of viral sequences, including RdRp-COVID, ORF lab-COVID, and E-genes from SARS-CoV-2, can be probably extended for viral detection from saliva (Qiu et al. 2020).

### Saliva: a clinical sample for COVID-19

**Fig. 11:**
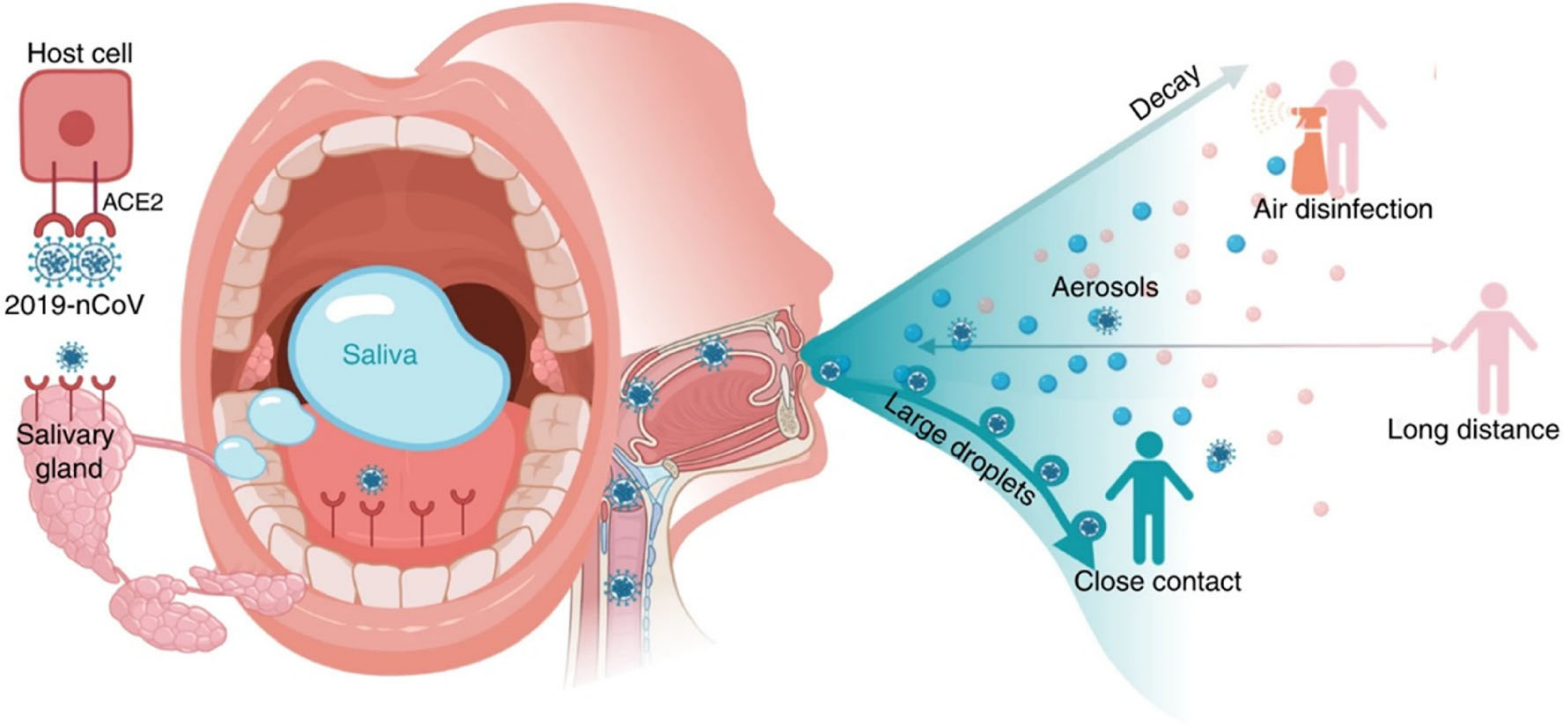
Potential diagnostic value of saliva and transmission of 2019-nCoV. Possibly binding to host-cell receptor of ACE2 expressed in salivary glands and tongue, 2019-nCoV is detected in saliva. Combined with infectious fluids from respiratory system, 2019-nCoV via large saliva droplets sets up short-distance transmission and hardly form long-distance aerosol transmission outdoors due to complicated physical and biological decay. Prevention of droplets formation, implementation of air disinfection, and blockage of droplets acquisition could possibly slow down 2019-nCoV dissemination (Reprinted from (Xu et al. 2020c) Under Creative Common License (http://creativecommons.org/licenses/by/4.0/)

Saliva is regarded as one of the primary transmission routes for COVID-19 due to its high predilection for spreading via sneezing coughing, breathing, and even through conversations (Fig. 11). In view of these, World Health Organization has urged the public to maintain ~1 m distancing from each other as a preventive measure to minimize COVID-19 transmission. This has been further revised to 2 m as per the guidelines issued by Centre for Disease control and prevention (CDC) (Bourouiba 2020). Viral detection based on saliva samples has been already employed efficiently for Zika and Ebola viruses (Niedrig et al. 2018; Khurshid et al. 2019). Viral RNA presence has been detected in the saliva of Zika infected subjects even after 29 days of symptom onset (Barzon et al. 2016). RT-PCR test results conducted on COVID-19 suspected patient’s samples have detected viral RNA presence up to the10^8^ copies/mL (Wang et al. 2004). Moreover, studies have also observed viral presence in saliva samples of patients which were found negative in nasopharyngeal aspirates (To et al. 2017). As per a recent study, viral presence has been detectable in the saliva of more than 90% patients suffering from SARS-CoV-2 infection (To et al. 2020a, b).

Reliability of saliva samples for COVID-19 diagnostics has been evaluated in a recent study, where PCR results of saliva were found positive for all the clinically diagnosed COVID-19 patients. The study also showed the viral presence in two subjects, even after the “gold standard” swab results turned out to be negative (Azzi et al. 2020). A study performed on SARS-COV-2 infected ferrets have also reported the appearance of viral RNA in the saliva, in addition to its presence in urine and feces (Kim et al. 2020). The amount of viral load in saliva has been at its maximum during the first week after symptom onset in SARS-COV-2 (To et al. 2020a, b). Epithelial cells present in the salivary glands have been recognized as the initial targets for SARS-COV-2, which might be the probable cause for the high viral load in saliva even at the early stages of infection (Liu et al. 2011). High expression of host cell receptors has been identified in the epithelial cells of oral mucosa (Xu et al. 2020a). The immunofluorescence staining study by Srinivasan et al. have demonstrated the expression of ACE2 in the epithelial cells of the oral mucosa (Srinivasan et al. 2020). The exfoliated epithelial cells in saliva have been also found with high expression of ACE2 receptor. A study reported on the previous SARS-COV epidemic patients has witnessed viral RNA upto ~6.38 × 10^8^ copies/ml in saliva, which was higher than that obtained from throat wash (Wang et al. 2004). The similarities and differences of saliva, between SARS-CoV and 2019-nCoV, are compared in a recent article, in terms of diagnosis value of saliva, direct invasion to oral tissues, and saliva droplet transmission, possibly explaining the faster transmission speed of 2019-nCoV (Xu et al. 2020b). The studies mentioned here clearly demonstrate that saliva can be a preferable diagnosis route for early detection of COVID-19 (Chojnowska et al. 2018; Xu et al. 2020c). Recently, US FDA has also given approval for accelerated emergency use of a saliva-based diagnostic kit for SARS-COV-2 (FDA 2020).

## Summary and future prospects

Despite all these advantages, the usefulness of saliva for routine clinical testing is not yet fully recognized. Even though, photonics technologies have found ubiquitous applications in diverse scientific fields, the clinicians and biomedical fraternity are still not fully conversant about the potential of photonics tools that can be used for routine applications in clinics. As indicated above, several studies have already pointed out the utility of saliva as a pool of biomarkers for the non-invasive detection of caries, periodontal diseases, oral cancer, lung cancer, prostate cancer and diabetes. Tables 1, 2, 3, 4, 5 to 6 summarize the recent research in photonics/optical spectroscopy based studies on saliva as a body fluid for clinical and other general applications.

**Table 1:** Selected references on use of photonics/optical spectroscopy-based studies on saliva for clinical and other applications. (Various cancers)

S. No	Method	Applications	Year and ref	Sensitivity/L.O.D
1	SERS	Oral Cancer: Saliva +Au nanoparticles. PCA of Raman	2020 [Fălămaș et al. 2020]	_
2	SERS	Oral Cancer healthy, mild and moderate dysplasia	2020 [(Muro et al. 2016a, b]	_
3	ATR-FTIR	Salivary exosomes;machine learning PCA &SVM	2020 [Su and Lee 2020]	_
4	ATR-FTIR	Salivary gland tumor (tumor mixus, TM)	2020 [Paluszkiewicz et al. 2020]	_
5	Raman	Oral Squamous cell carcinoma	2020 [Falamas et al. 2020]	93.6%
6	ATR-FTIR	Saliva for breast cancer	2020 [Ferreira et al. 2020]	94%
7	SERS	POC handheld. (S100P) mRNA in whole saliva; biomarker for oral cancer.	2019 [Han et al. 2019]	L.O.D in free solution:1.1 nM In VFC L.O.D:10nM
8	FTIR	Diagnosis of lung and breast cancers	2019 [Bel’skaya et al. 2019]	_
9	FTIR	Salivary exosomes; oral cancer (OC)	2019 [Zlotogorski-Hurvitz et al. 2019]	100%
10	General	Saliva for various cancers	2019 [Kaczor-Urbanowicz et al. 2019]	Lung cancer sensitivity 93.75%
11	ATR-FTIR	Oral cancer diagnostics. Spectra of Salivary Exosomes	2019 [Dekel et al. 2019]	100%
12	SERS	Liquid biopsy for various cancers.	2019 [Zhang et al. 2019]	miRNAs in liver cancer L.O.D 10fM
13	SERS	Ovarian cancer	2018 [(de Jesús Zermeño-Nava et al. 2018]	80%
14	Fluorescence	Oral pre-cancer	2018 [Kumar et al. 2018]	OSCC to normal 91%, OSMF to normal 92%
15	LIF	salivary proteins labelled with Cy3 fluorescent dye and detected by fluorescence	2018 [Liu et al. 2018]	60%
16	UV-Visible Absorption	Saliva in leukaemia	2018 [Joudah et al. 2018]	_
17	UV- Fluorescence	Steady and excited state kinetics; normal, oral premalignant and malignant subjects	2018 [Yuvaraj et al. 2018]	86.6%
18	SERS	Sialic acid (SA) in saliva; breast cancer Detection.	2017 [Hernández-Arteaga et al. 2017]	94%
19	Spectrophotometry	Salivary levels of zinc, iron and copper in Head and Neck Cancer	2017 [George et al. 2017]	_
20	SERS	Nasopharyngeal carcinoma NPC. SERS with PCA-LDA; non-invasive detection of NPC	2016 [Qiu et al. 2016]	86.7%
21	SPR	Recombinant human matrix metalloproteinases-9 (MMP-9); tumor progression and metastasis; saliva	2016 [Mohseni et al. 2016]	L.O.D : 8 pg/ml
22	SERS	Breast cancer	2015 [Feng et al. 2015]	Malignant 74.19%
23	HPLC-LIF	Various cancers	2015 [Patil et al. 2015]	L.O.D : femto-moles
24	AAS.	Copper, iron, zinc and manganese in oral submucous fibrosis	2015 [Okade et al. 2015]	_
25	Fluorescence	Oral Cancer	2014 [Yuvaraj et al. 2014]	Emission spectra sensitivity 85.7%
26	Fluorescence	Quantum Dots in Breast Cancer	2014 [Jokerst et al.]	L.O.D CEA: 0.02 ng/ml
27	HPLC-LIF	Salivary protein markers; Oral cancer	2018 [Zaorska et al. 2018]	79%
28	SPR	Carbohydrate antigen 15-3 (CA15-3) in saliva	2012 [Liang et al. 2012]	L.O.D : 2.5 U/ml
29	SERS	Saliva lung cancer	2011 [Li et al. 2011]	94%
30	Optical	Cancers	2008 [Tan et al. 2011]	L.O.D IL-8: 4 fM
31	SERS	5-fluorouracil: Chemotherapy drug; for solid tumors and colorectal carcinoma	2008 [Farquharson et al. 2008]	L.O.D: 2 μg/ml
32	SERS	5-Fluorouracil: Chemotherapy drug; for solid tumors and colorectal carcinoma	2005 [Farquharson et al. 2005]	L.O.D : 150 ng/ml
33	SPR	Interleukin-8(IL-8).Oropharyngeal squamous cell carcinoma (OSCC)	2005 [Yang et al. 2005]	L.O.D : 2.5 pM

**Table 2:** Selected references on use of photonics/optical spectroscopy-based studies on saliva for clinical and other applications. (Viral and other pathogen-related disorders)

1	SERS	Spike protein (S protein) of SARS-CoV-2 in human saliva	2020 [Jinglin et al. 2020]	_
2	Fluorescence	Coronavirus diagnosis	2020 [Bioptics World Editors 2020]	_
3	SERS, SPR	H1N1, HAdV and SARS-CoV-2	2020 [Cui and Zhou 2020]	SERS:50 and 10 pfu mL−1 SPR:0.22 pM
4	Raman	Detection of RNA viruses in saliva.	2020 [Desai et al. 2020]	92.5%
5	SERS	SARS-CoV-2	2020 [Yacaman et al. 2020]	_
6	SPR	Immunosensors: viruses, microbes, extracellular vesicles (EV)	2020 [Choi et al. 2020]	_
7	Interferometry	COVID- 19 saliva test	2020 [Wallace 2020]	L.O.D miRNA: attomole(am) range, 4 CFU/mL for whole pathogen detection.
8	SERS	Pandemic H1N1 (pH1N1) virus in human saliva.	2019 [Eom et al. 2019]	L.O.D : 1 PFU
8	SERS	Immunoassays for Detection of Botulinum Toxins	2019 [Kim et al. 2019a, b]	5.7 ng/ml(Type A L.O.D) 1.3 ng/ml(Type B L.O.D)
9	SERS	Bacteria sensing	2019 [Wang et al. 2019]	_
10	SERS	Avian influenza virus- H3N2	2017 [Sun et al. 2017]	10^2^ TCID_50_/mL
11	SERS	Pyocyanin (PYO), biomarker for Pseudomonas infections; in saliva.	2017 [Žukovskaja et al. 2017]	L.O.D: 0.5 μM
12	SPR	Avian influenza virus H5N1.	2012 [Bai et al. 2012	L.O.D : 0.128 HAU
13	Reflectometry	Influenza virus	2010 [Endo et al. 2010]	L.O.D: 1 ng/ml

**Table 3:** Selected references on use of photonics/optical spectroscopy-based studies on saliva for clinical and other applications. (Other disorders)

1	SERS	Saliva. Sjögren's syndrome (SjS)	2020 [Moisoiu 2020]	96.5%
2	ATR-IR	Periodontal Diseases	2020 [Beyer-Hans et al. 2020]	_
3	ATR-FTIR	Bio fluids including saliva; Review, clinical applications	2020 [Naseer et al. 2020]	_
4	Raman	Amyotrophic Lateral Sclerosis (ALS) diagnosis	2020 [Carlomagno et al. 2020]	_
5	SERS	Periodontitis, Sialic Acid (SA)	2019 [Hernández-Cedillo et al. 2019]	69.6%
6	SERS	To evaluate the activity of urease in saliva. Dental caries; helicobacter pylori infection	2019 [Hu et al. 2019]	L.O.D: 2.35 μg/ml
7	SERS	biomarkers of bronchial inflammation in the saliva of children with asthma	2019 [Zamora-Mendoza et al. 2019]	85%
8	ATR-FTIR	Chronic kidney disease	2019 [Rodrigues et al. 2019a, b]	92.8%
9	FTIR	Burning Mouth Syndrome	2019 [Rodrigues et al. 2019a, b]	_
10	FTIR	Neonatal sepsis using saliva	2019 [Yunanto et al. 2019]	_
11	ATR-FTIR	Multiple dental caries	2018 [Seredin et al. 2018]	_
12	Plasmonics	Hepatitis B	2017 [Riedel et al. 2017]	_
13	IR	Saliva in healthy, chronic, and aggressive periodontitis	2017 [Saranya 2017]	_
14	Plasmonics	Hepatitis B	2016 [Riedel et al. 2016]	_
15	FTIR	Differentiation of chronic and aggressive Periodontitis	2016 [Simsek Ozek et al. 2016]	82%
16	ICP OES & ICP MS	Periodontal diseases.	2016 [Herman et al. 2016]	L.O.D: 0.007 μg/l for Pb and 0.21 μg/l for Fe
17	Raman	Myocardial Infarction. Saliva spectroscopy; Multivariate analysis	2015 [Cao et al. 2015]	80.04%
18	FTIR	Saliva proteomic components in psoriatic and diabetic patients	2015 [Bottoni et al. 2015]	_
19	Fluorescence	Lithium in Bipolar disorder	2011 [Kim et al. 2011]	_
20	SPR	Mucin, Gingivitis, Periodontitis	2007 [Fernández-González et al. 2007]	_

**Table 4:** Selected references on use of photonics/optical spectroscopy-based studies on saliva for clinical and other applications. (Drugs)

1	SERS	Detection of methamphetamine in saliva and urine	2020 [Hong et al. 2020]	L.O.D: nM
2	SERS	Detection of morphine	2020 [Li et al. 2020]	2.4×10^−4^ *ng*/*ml*
3	SERS	Tetrahydrocannabinol (THC) in human plasma and saliva	2019 [Sivashanmugan et al. 2019a, b]	L.O.D: 1pM
4	SERS	Tetrahydrocannabinol (THC) in saliva	2019 [Sivashanmugan et al. 2019a, b	L.O.D: 10 ppm
5	SERS	Codeine and Fentanyl; in saliva and blood	2019 [Shende et al. 2019]	L.O.D: 0.5 μg/ml
6	SERS	Plasmonic capsules based SERS: methamphetamine (MA), in human saliva	2019 [Su et al. 2019]	67.4%
7	SERS	Ultra-traces of cocaine	2018 [D’Elia et al. 2018]	L.O.D: 10ng/ml
8	SERS	Detection of drug-related biomarkers	2015 [Yang et al. 2015]	L.O.D: 100 nM
9	SERS	Cocaine; in saliva.	2015 [Dana et al. 2015]	L.O.D: 25 ng/ml
10	SERS	Illicit Drugs; in saliva	2014 [Shende et al. 2014]	_
11	IR	Cocaine in saliva	2014 [Hans et al. 2014]	L.O.D: 100 ng/ml
12	SERS	methamphetamine	2013 [Andreou et al. 2013]	L.O.D: 10 nM
13	ATR-IR	Cocaine in saliva	2012 [Hans et al. 2012]	_
14	SERS	Drugs of abuse; in saliva	2011 [Inscore et al. 2011]	L.O.D : 1 ppm
15	SERS	Cocaine in saliva	2011 [Farquharson et al. 2011]	L.O.D: 50 ng/ml
16	SPR	pharmaceutical compounds cocaine and MDMA(3,4-methylenedioxy-methamphetamine)	2010 [Sonny et al. 2010t	_
17	Raman	Saliva of narcotic users	2009 [Anyu et al. 2009]	_
18	SPR	Antiepileptic drug (AED) phenytoin	2007 [Fu et al. 2007]	L.O.D: 50 nM
19	SERS	Drugs and metabolites; in saliva	2005 [Shende et al. 2005]	_

**Table 5:** Selected references on use of photonics/optical spectroscopy-based studies on saliva for clinical and other applications. (Other abnormal conditions)

1	LSPR	Cortisol, stress biomarker	2020 [Jo et al. 2020]	L.O.D: 0.1 nM
2	UV Absorption	Stress biomarkers in different body fluids	2019 [Ray and Steckl 2019]	L.O.D of cortisol: 0.5 μM
3	SERS, LIF, SPR	Diseases, drugs, general health	2019 [Ilea et al. 2019]	_
4	FTIR	Real-time checking of response to stress	2016 [Caetano Júnior et al. 2016]	_
5	SPR	Hormones (Cortisol, Testosterone)	2014 [Stevens et al. 2014, Mitchell and Lowe 2009]	L.O.D of cortisol: 1 ng/ml L.O.D of Testosterone: 29 pg/ml
6	SPR	Cortisol	2014 [Tahara et al. 2014]	L.O.D: 38 ppt
7	SPR	Cathespin G, Immune response	2011 [Gorodkiewicz et al. 2011]	L.O.D: 0.12 ng/ml
8	FTIR	Saliva of normal and diabetic pregnant women in each trimester.	2011 [Sultana et al. 2011]	_
9	SPR	Cathespin D	2010 [Gorodkiewicz and Regulska 2010]	L.O.D: 0.12 ng/ml
10	ATR-FTIR	Saliva biomarker; exercise induced stress. Real-time checking of response to stress.	2010 [Khaustova et al. 2010]	_
11	SPR	Cortisol and cortisone	2009 [Frasconi et al. 2009]	L.O.D: 10 μg/l

**Table 6:** Selected references on use of photonics/optical spectroscopy-based studies on saliva for clinical and other applications. (Other applications)

1	IR	Age and gender	2020 [Bel’skaya et al. 2020]	_
2	AAS	Metal residuals in saliva: orthodontic Appliances	2020 [Curro, and Bilello 2020]	L.O.D : μg/l
3	IR	Spectral features in tobacco smoking, periodontal diseases, and gender.	2019 [Derruau et al. 2019]	_
4	AAS	Release of nickel, chromium, and zinc in saliva; Use of orthodontic appliances	2019 [Quadras et al. 2019]	_
5	SERS	Effects of low-dose irradiation on saliva	2019 [Colceriu-Șimon et al. 2019]	_
6	LIBS	Calcium in saliva	2019 [Zahroh et al. 2019]	_
7	IR	Saliva composition	2018 [Bel’skaya et al. 2018]	_
8	ATR-FTIR	Forensic, body fluids, including Saliva	2018 [Takamura et al. 2018]	_
9	SPR	ABH antigen detection in saliva.	2017 [Peungthum et al. 2017]	_
10	LSPR	Wine astringency. Interaction of red wine and saliva	2017 [Guerreiro et al. 2017]	L.O.D : 1 μmol/L
11	LSPR	Monitoring the pH of saliva	2017 [Luo et al. 2017]	wavelengths of 665 nm and 785 nm—the sensitivities were 0.0299 a.u./pH (a.u. = arbitrary unit) with a linear range of pH = 5–8 and 0.0234 a.u./pH with linear range of pH = 2–8, respectively
12	FTIR	Effect of smoking cessation on saliva	2017 [Rodrigues et al. 2017]	_
13	TRLFS	ingestion of radioactive contaminants, time-resolved laser-induced fluorescence spectroscopy	2017 [Barkleit et al. 2017]	_
14	Raman	Forensic body fluid identification	2016 [Muro et al. 2016]	_
15	Raman	Sex determination based on saliva spectrum; forensic	2016 [Muro et al. 2016a, b]	92%
16	IR, Raman	Forensic analysis	2015 [Zapata et al. 2015]	_
17	SPRi-MS	Protein biomarkers salivary α-amylase and lysozyme.	2015 [Musso et al. 2015]	_
18	Raman	Forensic analysis	2010 [Virkler and Lednev 2010]	_

As is evident from Tables 1, 2, 3, 4, 5 to 6, saliva is a highly suitable body fluid for screening of not only clinical aspects but also in many other areas like problems in the use of dental accessories, influence of metallic elements in conditions like malnutrition, evaluation of effects of dangerous external invasions like exposure to nuclear radiation or chemical/biological agents. Amongst all biofluids, saliva is easiest to collect and the collection is non-invasive. As seen from the various studies discussed above, saliva shows numerous biomarkers, in many abnormal states in the living system. As of now, clinicians are completely relying on laboratory-based RT-PCR assays of saliva for diagnostic applications, especially in viral detection methods, even though false positives/negatives are a major issue in this technique. Photonics-based techniques can be of great interest in developing reliable technologies for the detection/identification of viruses like SARS-CoV-2. Use of saliva as the clinical specimen has several possibilities including detection of virions, identification of specific antibodies against Virus and identification of markers of the non-specific immune response in saliva. Saliva can be a reliable source for new disease biomarkers and/or understanding of pathways involved in diseases. Even though established techniques such as two dimensional (2D)-gel electrophoresis, matrix assisted laser desorption ionization (MALDI) and surface-enhanced laser desorption/ionization (SELDI)-mass spectroscopy, and capillary electrophoresis (CE) can acquire protein, or other markers’ profiles from clinical samples, these suffer from several drawbacks. Two-dimensional polyacrylamide gel electrophoresis (2D-PAGE) is time consuming, require elaborate sample preparation and in addition to the presence of minor proteins at trace concentrations can be masked by major proteins. Surface-enhanced laser desorption/ionization (SELDI)-Time of Flight (TOF) mass spectroscopy (MS) or other mass spectrometric methods require bulky instrumentation and qualified professionals for operation, making them unsuitable for large-number-screening at multiple locations in a short period, as will be required in pandemic situations.

Advanced research in salivaomics can play a pivotal role in screening and early detection of many diseases as well as disease-causing agents like virions and microbes (Farah et al. 2018; Liu et al. 2018; Shah 2018; Gug et al. 2019; Pathiyil and Udayasankar 2019). In spite of this possibility, acceptance of salivary markers for clinical applications, like regular screening and diagnosis, follow-up in therapy, or point-of-care/point of use applications, like screening and environmental monitoring in situations like the COVID pandemic, has not happened yet. The possible reason for this seems to be the fact that all such studies have been carried out by only one or two groups with specific sample classes, and has not been validated over large groups of cohorts to ensure adequate representation of sample/disease heterogeneity. What is urgently required to make salivaomics an effective diagnostic approach is a close collaboration between Academia with multi-disciplinary expertise—Medical, Physical and Engineering sciences—and Regulatory and Funding Agencies to standardize the technologies involved, through extended research by multiple groups in a concerted manner. Another essential requirement is that the markers observed in saliva, mainly proteins, enzymes and DNA/RNA species should be studied further by bio-chemical and spectroscopic methods, including high-sensitivity mass spectrometry, from the point of view of correlating them to one another, to provide fundamental information about the different bio-molecular interactions, stages and variations in the disease, so that their validity as suitable markers can be further confirmed.

The lack of such a concerted approach has slowed the pace of standardization and routine use of saliva for clinical applications, leaving its potential for clinical applications unutilised. At present, photonics techniques has been in the forefront for providing solutions to medical community for early detection of diseases and prognosis of treatment. It may be appropriate to mention another major aspect of photonics technology highly relevant in the present context. Photonics systems operate through interaction of photons with matter, a physically non-contact process for the investigators. This allows the observations of materials by remote sensing, without the need for the observer to be in close proximity of the subject or material which is being studied. With current advances in lasers, spectrographs and imaging, and information technology, it is thus possible to use photonics-based portable instrumentation in applications needing observation of, subjects in intensive care units (ICUs), neonatal, differently abled and isolation/quarantine conditions or environmental samples from suitable distances. Finally, it has to be noted that current photonics systems enable observation of multi-modal (LIF+ RAMAN, LIF+ LIBS, Raman + LIBS, Absorption +Fluorescence) spectral observations on a same given sample in a single (e.g. resonance Raman and fluorescence, absorption and fluorescence) measurement, or one after the other without any change in the sample characteristics. Similarly, multi-wavelength spectral studies also can be done. Both these techniques can provide multiple data sets on a given sample, enabling more accurate data processing by machine-learning and artificial intelligence (AI) methods, so that specificities and sensitivities can be improved over the current 80–90% level. Such instrumentation can carry out multi-modal spectral data acquisition, providing unambiguous identification and classification (Dhanada et al. 2020). This is especially of great advantage for clinical samples because all clinical samples, usually, are suitable for both fluorescence and Raman spectral studies, and current laser systems can give excitation wavelengths in the UV region. This allows a single laser-spectrometer combination system which can simultaneously give fluorescence and Raman, without any mutual interference, since the fluorescence form many physiological samples usually comes well-after 300 nm, while the Raman scattered radiation, which falls in the 100-3000cm^-1^ region after the excitation wavelength comes before the fluorescence, so that the two spectra show no mutual interference (Lednev 2019).

It is seen that remote observation of multiple spectral information can be highly useful in present circumstances, when many people have to be tested for their health status, keeping social distances. It is possible to monitor the health status of an individual by looking at even exposable organ surfaces like skin, for such screenings in remote mode, since the laser powers for LIF are at safe levels for exposure. In future, one can thus expect considerable research in bio-medical applications with such advanced multi-modal, multi-excitation techniques, applicable in vivo with accessible organs (skin, oral cavity) and easily accessible samples like saliva and breath.

## Conclusion

The present situation arising from the COVID pandemic has highlighted the need of universal screening technologies with fast, sensitive devices for in situ screening of large cohorts at multiple locations like airports or other areas where large numbers assemble for various purposes. Early diagnosis is also necessary in many situations in hospitals, clinics, test laboratories, and even at home. Portable/handheld screening devices comprising of Photonics tools coupled with machine learning and artificial intelligence, can be developed. Powerful optical techniques available at present have demonstrated the capabilities to detect, diagnose and stage different kinds of infections and non-infections disease with very high sensitivity and specificity with minimal sample handling. Such systems can be easily adapted for the diagnosis of COVID-19 kind of pandemic. The photonics of saliva offer an area of large possibilities for such applications. A major noteworthy factor in using the photonics-based methods is the possibility of the multi-modal approach. In almost all work so far, attempts have been concentrated on using only a single modality, like Raman, LSPR, LIF, and IR. The main consequence of such mono-modal approach has been results with specificities and sensitivities in the range of 80–90%. But it is highly likely that a multi-modal approach, say, combination of two or three spectroscopic data sets with AI/Machine Learning (ML) techniques should be able to give still better results. It is therefore, highly desirable to carry out studies to develop multi-modal spectral methods, especially where large number of samples have to be tested in short time intervals, since the multi-modal approach does not involve any additional time and same sample can be studied in different data analyses methods.
